# Effects of Glucomannan on Cardiometabolic Parameters in Adults With Insulin Resistance‐Related Conditions: A Systematic Review and Dose–Response Meta‐Analysis

**DOI:** 10.1002/fsn3.72460

**Published:** 2026-10-08

**Authors:** Mostafa Shahraki Jazinaki, Hossein Rafiei, Hossein Bahari, Naseh Pahlavani

**Affiliations:** ^1^ Department of Nutrition, Faculty of Medicine Mashhad University of Medical Sciences Mashhad Iran; ^2^ Student Research Committee Mashhad University of Medical Sciences Mashhad Iran; ^3^ Department of Nutrition and Dietetics Saint Louis University Saint Louis Missouri USA; ^4^ Social Determinants of Health Research Center Torbat Heydariyeh University of Medical Sciences Torbat Heydariyeh Iran; ^5^ Health Sciences Research Center Torbat Heydariyeh University of Medical Sciences Torbat Heydariyeh Iran

**Keywords:** blood pressure, glucomannan, glycemic control, insulin resistance, lipid profile, obesity

## Abstract

Inconsistent findings regarding the impacts of glucomannan intake on health‐related outcomes have been reported in different health conditions. Therefore, this meta‐analysis aimed to assess the impacts of glucomannan consumption on cardiometabolic parameters in adults with insulin resistance‐related conditions. PubMed, Scopus, and Web of Science databases were comprehensively searched up to March 2025 to find eligible studies. Overall effect sizes were calculated according to the random‐effects model and expressed as weighted mean differences (WMDs) with 95% confidence intervals (95% CIs). Heterogeneity among the included trials was assessed by performing Cochran's Q test and measured by the *I* ^2^ statistic. Eleven eligible trials were included in this review. Meta‐analysis revealed that glucomannan significantly reduced fasting blood glucose (FBG) (WMD: −7.02 mg/dL, 95% CI: −13.56, −0.49, *p* = 0.04); however, this effect was no longer statistically significant under the alternative method for the correlation assumption. Meanwhile, the effects of glucomannan intake on lipid profile, obesity indices, and blood pressure were not significant. Exploratory subgroup analyses suggested reductions in SBP, total cholesterol, and low‐density lipoprotein cholesterol (LDL‐C) in studies using higher glucomannan doses (> 3000 mg/day) or shorter intervention durations (≤ 4 weeks); however, these subgroup findings should be interpreted cautiously. This meta‐analysis suggests that glucomannan intake may reduce FBG levels in insulin‐resistance‐related conditions. However, due to a limited number of eligible trials, more high‐quality randomized controlled trials are needed to reach firm conclusions regarding the effectiveness of this intervention in different health conditions and to explore optimal dosage and duration for each therapeutic purpose.

## Graphical abstract

In the primary analysis, glucomannan supplementation was associated with a significant reduction in fasting blood glucose in adults with insulin resistance‐related conditions; however, this effect was not significant under the alternative correlation assumption. No significant overall effects were observed on lipid profile, obesity indices, or blood pressure. Exploratory subgroup analyses suggested reductions in systolic blood pressure, total cholesterol, and LDL‐C with higher doses (> 3000 mg/day) or shorter intervention durations (≤ 4 weeks).

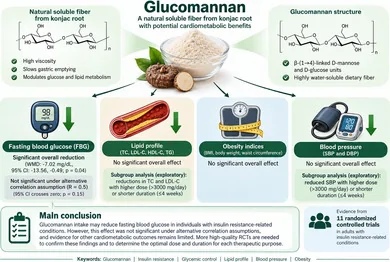

## 1 Introduction

Insulin resistance, defined as an impaired response of insulin‐target tissues to normal insulin levels, is a major global health concern, with a reported prevalence ranging from 15.5% to 46.5% worldwide (Lee et al. 2022; Fahed et al. 2020). In the United States, recent National Health and Nutrition Examination Survey (NHANES) data show that about 40% of adults aged 18–44 years have insulin resistance, even without overt diabetes, highlighting its critical role in metabolic health (Parcha et al. 2022). Insulin resistance is recognized as a risk factor for Type 2 diabetes (T2D) (Do et al. 2010), cardiovascular diseases (CVDs) (Mir et al. 2025), and high blood pressure (Maffeis et al. 2010). Also, it is a key factor in the development of several chronic conditions, including polycystic ovarian syndrome (PCOS), nonalcoholic fatty liver disease (NAFLD), metabolic syndrome, hypertension, T2D, and accelerated atherosclerosis (Lebovitz 2001; Zhao et al. 2023). The pathogenesis of insulin resistance is multifactorial, and age‐dependent body changes, obesity, genetic predisposition, certain medications, chronic inflammation, lipotoxicity, and hypoxia can all disrupt insulin signaling pathways (Zhao et al. 2023; Rizvi and Rizzo 2024; De Luca and Olefsky 2008). In addition, excess adiposity, especially visceral fat, increases lipolysis in the adipose tissue and consequently leads to free fatty acid flux to other tissues, worsening insulin sensitivity (Kahn and Flier 2000). Lifestyle interventions, including weight loss, increased physical activity, and medications such as GLP‐1 receptor agonists or insulin sensitizers, remain among the key approaches for managing insulin resistance (Desouza et al. 2001; Abdul‐Ghani et al. 2024; Granberry and Fonseca 1999; Scheen 1997). However, there is growing interest in complementary nutritional strategies, such as dietary fiber supplementation, to enhance insulin sensitivity (Dong et al. 2019).

Glucomannan, a viscous, soluble, and fermentable fiber from the konjac root ( *Amorphophallus konjac* ), or elephant yam, native to Asia, has attracted attention for its potential metabolic benefits (Lyon and Reichert 2010; Keithley et al. 2013; Keithley and Swanson 2005). The viscosity of this fiber is five times higher than that of other fibers, such as β‐dextran and guar gum, which may help delay gastric emptying, slow carbohydrate absorption, and promote satiety (Vuksan et al. 2000). Previous meta‐analyses have reported inconsistent findings. A meta‐analysis by Sood et al. in 2008 showed that glucomannan significantly decreased body weight, fasting blood glucose (FBG), triglycerides (TG), total cholesterol (TC), and low‐density lipoprotein cholesterol (LDL‐C), with no significant effect on high‐density lipoprotein cholesterol (HDL‐C) (Sood et al. 2008). In another study conducted by Musazadeh et al. (2024), a significant reduction in TC, LDL‐C, HDL‐C, and apolipoprotein (Apo) B1 level was observed following glucomannan supplementation, while changes in TG, Apo A1, APO‐B/A1 ratio, and LDL‐C/HDL‐C ratio were not significant. In addition, a meta‐analysis conducted by Haijun et al. (2025) showed that glucomannan supplementation led to a significant decrease in TC and LDL‐C levels compared to the control groups, while it had no significant impact on TG and HDL‐C levels.

According to the inconsistency in the effectiveness of glucomannan supplementation on cardiometabolic parameters in adults, it's important to assess the impact of glucomannan intake across various populations with different health conditions may help to understand the effectiveness of glucomannan supplementation on cardiometabolic parameters.

In the meta‐analysis that was conducted on T2D patients, Zhang et al. observed significant improvements in body weight, glycemic (FBG, P2hBG, and fasting insulin), and lipid parameters (TC and LDL‐C) among individuals with T2D, though effects on body weight, TG, HDL‐C, systolic blood pressure (SBP), and diastolic blood pressure (DBP) remained nonsignificant (Zhang et al. 2023). In another meta‐analysis that included studies conducted on individuals with overweight and obesity, glucomannan supplementation significantly decreased body weight in comparison to the control group (Mohammadpour et al. 2020).

However, these studies did not include trials that enrolled participants with other conditions related to insulin resistance. In addition, to date, no systematic review and meta‐analysis has comprehensively assessed the impacts of glucomannan intake on a broad range of cardiometabolic outcomes in adults with diverse insulin resistance‐related conditions. Therefore, this systematic review and meta‐analysis aimed to comprehensively investigate the effect of glucomannan consumption on cardiometabolic parameters in adults with conditions associated with insulin resistance.

## 2 Materials and Methods

The Preferred Reporting Items for Systematic Reviews and Meta‐Analysis (PRISMA 2020) framework was the basis of this review (Page et al. 2021). The protocol for conducting this research is registered in the PROSPERO database with the following registration ID: CRD420251055106.

### 2.1 Search Strategy

Scopus, Web of Science, and PubMed were comprehensively searched up to March 2025 to identify relevant interventional studies with an appropriate control group investigating the effects of glucomannan intake on cardiometabolic parameters in adults with insulin resistance‐related conditions. The following free‐text and MeSH terms were used to construct the search strategy (“glucomannan” OR “ *Amorphophallus konjac* ” OR “konjac” OR “konnyaku” OR “devil's tongue” OR “elephant foot”) AND (“diabetes” OR “polycystic ovary syndrome” OR “PCOS” OR “metabolic syndrome” OR “syndrome X” OR “insulin resistance syndrome” OR “obese” OR “obesity” OR “overweight” OR “insulin resistance” OR “prediabetes” OR “hyperinsulinemia” OR “glucose intolerance” OR “hyperglycemia”) AND (“intervention” OR “randomized” OR “placebo” OR “clinical trial” OR “randomized controlled trial” OR “randomized clinical trial” OR “RCT” OR “cross‐over” OR “parallel”).

In PubMed, all glucomannan‐related terms were searched as free‐text terms in the Title/Abstract field. Condition‐related terms were searched using both free‐text terms and the MeSH terms “Diabetes Mellitus,” “Obesity,” “Overweight,” and “Metabolic Syndrome.” The study‐design component included free‐text terms and the MeSH term “randomized controlled trial.” Complete database‐specific search strategies, including field tags and Boolean operators, are provided in Table S1.

Google Scholar was also searched manually. No restriction was applied to the publication date; however, only studies published in English were considered eligible. To minimize the possibility of missing relevant studies, the reference lists of the included trials were also manually screened. Records identified through the database searches were independently screened for eligibility by two authors (M.Sh.J. and H.B.).

### 2.2 Eligibility Criteria

In this review, the eligibility criteria were designed according to the PICOS framework as follows (Population: Adults with insulin resistance states, including overweight and obesity, polycystic ovarian syndrome (PCOS), insulin resistance/hyperinsulinemia, hyperglycemia, prediabetes/glucose intolerance, or T2D; Intervention: Glucomannan intake; Control: not consuming glucomannan or receiving placebo; Outcomes: cardiometabolic parameters; and Study design: intervention studies with a control group; Amir‐Behghadami and Janati 2020).

Eligible studies were required to be interventional trials with an appropriate control group, including adults (≥ 18 years) with insulin resistance‐related disorders, to have glucomannan intake in the intervention group, and to report mean changes and standard deviations (SDs) for relevant cardiometabolic outcomes or provide measures at baseline and the end of intervention to calculate these values.

The exclusion criteria in this review include animal studies, observational studies (case–control, cohort studies, and cross‐sectional), acute postprandial intervention studies, ecological studies, short communications, letters to the editors, trials that did not report changes in cardiometabolic outcomes, studies without an appropriate control group, combination interventions, and studies performed on non‐adults (age < 18).

### 2.3 Data Extraction

The extraction of the relevant data was done by two independent authors (M.Sh.J and H.B), from each included study. The following data were extracted: first author's name, country of origin of research, publication year, study design, the characteristics of participants including health status, mean body mass index (BMI), and mean age, intervention features with glucomannan (type, dosage, and duration), measures taken in the control group, and mean differences with their SDs for each mentioned outcome in each group. Any disagreement was discussed until a consensus was reached.

### 2.4 Risk of Bias Assessment

The Cochrane risk‐of‐bias tool (ROB2) was used to assess the risk of bias in the eligible trials by two independent researchers (H.B. and M.Sh.J.; Sterne et al. 2019, Higgins et al. 2019). This tool assesses the risk of bias in five key domains, including bias in the selection of reported results, bias in outcome measurement, bias resulting from deviations from intended interventions, bias arising from the randomization process, and bias due to missing data.

### 2.5 Data Synthesis and Statistical Analysis

All analyses conducted in this meta‐analysis were conducted using STATA software version 17. The pooled effect size in this meta‐analysis was calculated as a weighted mean difference (WMD) with a 95% confidence interval (95% CI). The overall effect size for each outcome was estimated using mean changes and their SDs during the study in the intervention and control groups according to the random‐effects model (Dersimonian and Laird 1986). In cases where changes were not reported directly, they were calculated by subtracting measures of outcomes at baseline from those at the end of the intervention. Also, SDs for mean changes could be estimated by using the following formula: square root [(SDbaseline)^2^ + (SDfinal)^2^ − (2 × *R* × SDbaseline × SDfinal)] (Borenstein et al. 2021). A correlation coefficient (R) of 0.8 was assumed. By using the method of Hozo et al. (2005), 95% confidence intervals (CIs), interquartile ranges (IQRs), and standard errors (SEs) were converted to SDs. The heterogeneity among the eligible trials was assessed by carrying out Cochran's *Q* test and measured according to the interpretation of the *I*‐squared statistic (*I* ^2^) (Higgins et al. 2003). *I* ^2^ > 40% or *p*‐value < 0.05 was deemed significant heterogeneity between included trials. Subgroup analyses were performed to find possible sources of heterogeneity based on the following predefined criteria (Higgins and Thompson 2002; Bahari et al. 2024): Country (USA, Canada, and others), study design (parallel and cross‐over), gender (both sexes, only females and only males), age (≤ 50 and > 50 years), trial duration (≤ 4 and > 4 weeks), glucomannan dosage (≤ 3000, > 3000 mg/day, and not reported), health status (T2D, overweight/obesity, and others), and mean baseline BMI (25–29.9, and > 30). Sensitivity analyses were conducted using two complementary approaches: (1) a leave‐one‐out analysis in which each effect size was sequentially excluded to evaluate its influence on the pooled estimate (Tobias 1999), and (2) an analysis in which the assumed correlation coefficient (*R*) was maintained at 0.8 for parallel‐group studies but reduced to 0.5 between baseline and final measurements in crossover studies.

The publication bias was evaluated by visual interpretation of funnel plots and performing Egger's regression test (Egger et al. 1997). Meta‐regression and fractional polynomial modeling were performed to assess the linear and non‐linear relationships between intervention features (dosage and duration) and outcome level changes, respectively (Dehkordi et al. 2024; Monfared et al. 2023; Mousavi et al. 2020). In all performed analyses, *p*‐value < 0.05 was identified as the statistical significance level.

### 2.6 GRADE Analysis

According to the GRADE (Grading of Recommendations Assessment, Development, and Evaluation) approach, the levels of evidence certainty were assessed by two authors (M.Sh.J and H.B), independently (Guyatt et al. 2008). This framework evaluated the limitations of the included trials across five domains: risk of bias, indirectness, inconsistency, publication bias, and imprecision. The overall quality was classified into four levels: very low, low, moderate, and high. Any disagreements were resolved through discussion until consensus was reached.

### 2.7 AI Use Statement

All aspects of this study, including the study design, data analysis, interpretation of the findings, and preparation of the manuscript, were conducted by the authors. ChatGPT (OpenAI) was used solely to improve the language, clarity, and readability of the manuscript. The authors take full responsibility for the content, interpretation of the findings, and final version of the manuscript.

## 3 Results

### 3.1 Study Selection

Out of 289 papers found, 103 duplicate records were removed. Among the 186 screened studies, 145 were irrelevant for this review topic and were excluded. Two papers could not be retrieved for full‐text assessment (Biancardi et al. 1989; Chavez et al. 1998). Reading the full text of the 39 remaining studies, 28 did not meet our inclusion criteria due to being animal studies (*n* = 3), conducting combination therapy (*n* = 4), intervention on non‐adults (*n* = 3), protocol article (*n* = 1), non‐English papers (*n* = 2), studies with no appropriate control group (*n* = 3), conference abstract (*n* = 2), review article (*n* = 4), postprandial studies (*n* = 4), and not reporting desired data (*n* = 2). Finally, 11 trials were eligible for inclusion in this systematic review (Walsh et al. 1984; Vuksan et al. 1999, 2000; Chen et al. 2003; Yoshida et al. 2006; Chearskul et al. 2007; Wood et al. 2007; Li et al. 2022; Kaats et al. 2015; Jenko Pražnikar et al. 2023; Fernandes et al. 2023) (Figure 1).

**FIGURE 1:**
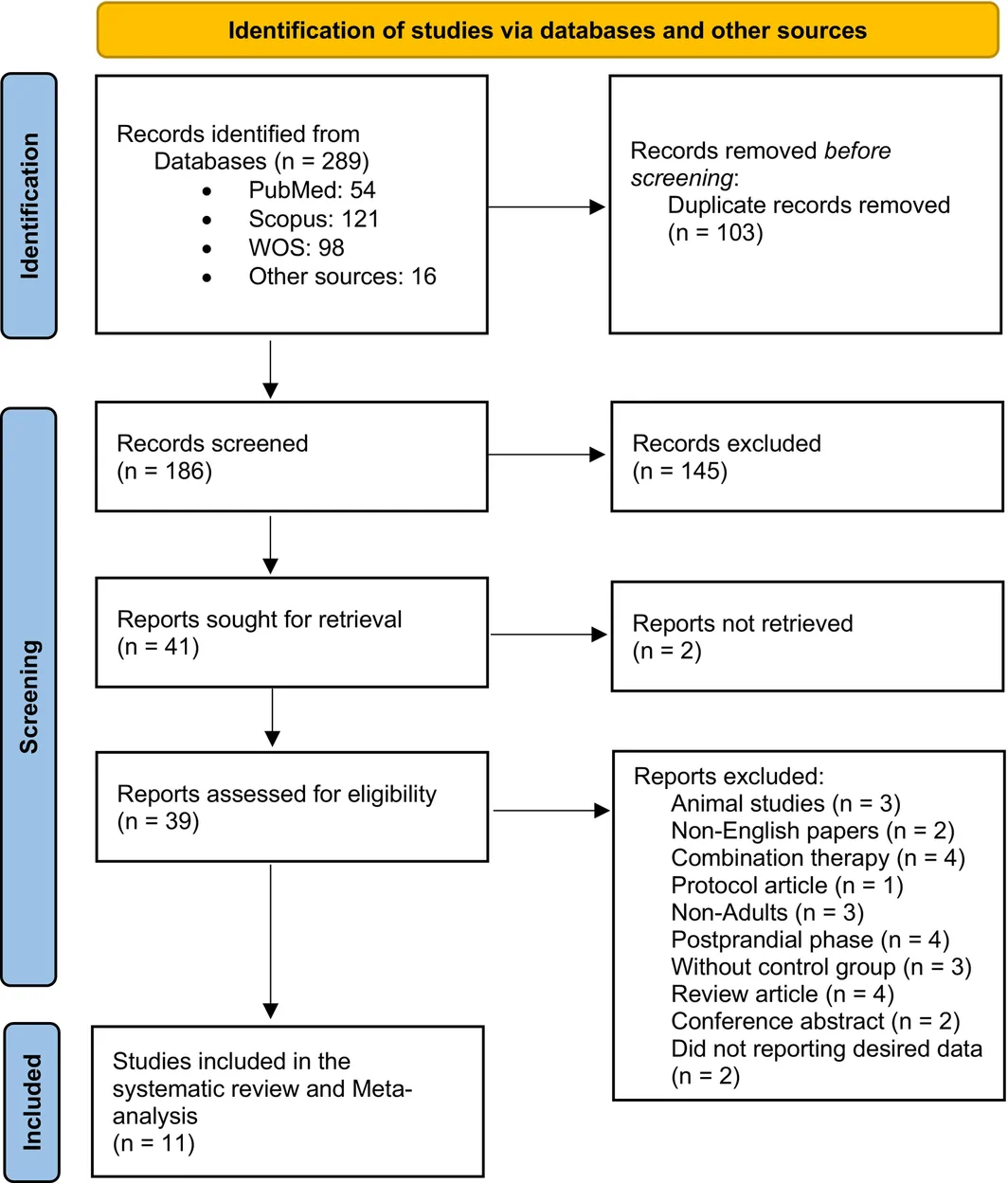
Flow chart of the study selection process for inclusion in this meta‐analysis.

### 3.2 Study Characteristics

Eligible studies were published between 1984 (Walsh et al. 1984) and 2023 (Fernandes et al. 2023; Jenko Pražnikar et al. 2023). Among the 11 included trials, three were conducted in USA (Walsh et al. 1984; Wood et al. 2007; Kaats et al. 2015), three in Canada (Vuksan et al. 1999, 2000; Yoshida et al. 2006), and one in each the, China (Li et al. 2022), Slovenia (Jenko Pražnikar et al. 2023), Brazil (Fernandes et al. 2023), Thailand (Chearskul et al. 2007), and Taiwan (Chen et al. 2003). All of the included trials were conducted on both sexes, except two studies (Wood et al. 2007; Fernandes et al. 2023), which were conducted on only males and only females, respectively. However, in one included trial, the genders of the population was not reported (Walsh et al. 1984). Out of 11 included trials, five had a cross‐over design (Vuksan et al. 1999, 2000; Chen et al. 2003; Yoshida et al. 2006; Chearskul et al. 2007), and six had a parallel design (Fernandes et al. 2023; Jenko Pražnikar et al. 2023; Kaats et al. 2015; Li et al. 2022; Walsh et al. 1984; Wood et al. 2007). The sample size of studies varied between 11 (Vuksan et al. 1999, 2000) and 80 participants (Kaats et al. 2015). The mean age and BMI of the population were between 30.8 (Li et al. 2022) and 64.2 years (Chen et al. 2003), and 25.5 (Chen et al. 2003), and 31.3 kg/m^2^ (Li et al. 2022). However, in the two included studies, the mean BMI of participants was not reported (Vuksan et al. 1999; Walsh et al. 1984). The population of included studies was individuals with T2D (Yoshida et al. 2006; Vuksan et al. 1999; Chen et al. 2003; Chearskul et al. 2007), overweight or obesity (Walsh et al. 1984; Wood et al. 2007; Kaats et al. 2015; Li et al. 2022; Jenko Pražnikar et al. 2023; Fernandes et al. 2023), or insulin resistance syndrome (Vuksan et al. 2000). The duration of included studies ranged from 3 (Vuksan et al. 1999, 2000) to 12 weeks (Wood et al. 2007). Dosage of received glucomannan was between 3000 (Walsh et al. 1984; Wood et al. 2007; Chearskul et al. 2007; Kaats et al. 2015; Jenko Pražnikar et al. 2023) and 10,000 mg/day (Yoshida et al. 2006). However, one trial did not report the dosage of the received glucomannan (Li et al. 2022). Also, in the two included studies that used Konjac‐mannan fiber–enriched biscuits, the glucomannan content was reported as 0.7 g/100 kcal (Vuksan et al. 1999), and 0.5 g/100 kcal of dietary intake (8–13 g/day; Vuksan et al. 2000). The summary of characteristics of the included effect sizes is provided in Table 1.

**TABLE 1:** Characteristics of included studies in this meta‐analysis.

Studies	Country	Study design	Participant health status	Sample size and Sex	Sample size		Trial duration (Week)	Means age		Means BMI		Intervention	
					IG	CG		IG	CG	IG	CG	Intervention type and dosage	Control group
Walsh et al. (1984)	USA	Parallel, R, PC, DB	Obesity	20 NR	10	10	8	NR	NR	NR	NR	Glucomannan 3000 mg/day	Placebo
Vuksan et al. (1999)	Canada	Cross‐over, R, PC, DB	T2D	11 B	11	11	3	60.5	60.5	NR	NR	Konjac‐mannan fiber–enriched biscuits 0.7 g/100 kcal	Wheat bran fiber biscuits (Placebo)
Vuksan et al. (2000)	Canada	Cross‐over, R, PC, DB	Insulin Resistance Syndrome	11 B	11	11	3	55	55	28	28	Konjac‐mannan fiber–enriched biscuits 0.5 g/100 kcal (8–13 g/day)	Wheat bran fiber biscuits (Placebo)
Chen et al. (2003)	Taiwan	Cross‐over, R, PC, DB	T2D	21 B	21	21	4	64.2	64.2	25.5	25.5	Konjac powder 1500 mg/day	Food‐grade corn starch (Placebo)
Yoshida et al. (2006)	Canada	Cross‐over, R, PC, DB	T2D	13 B	13	13	3	56.81	56.81	30.96	30.96	Glucomannan 10,000 mg/day	Placebo
Chearskul et al. (2007)	Thailand	Cross‐over, R, PC, SB	T2D	20 B	20	20	4	51.2	51.2	26.26	26.26	Glucomannan 3000 mg/day	Placebo
Wood et al. (2007)	USA	Parallel, R, PC, DB	Overweight and obese	30 M	15	15	12	20–69 (mean age of both groups: 38.8)	20–69 (mean age of both groups: 38.8)	25–35 (mean age of both groups: 29.7)	25–35 (mean age of both groups: 29.7)	Glucomannan 3000 mg/day	Placebo
Kaats et al. (2015)	USA	Parallel, R, PC, DB	Overweight	80 B	38	42	8.5	47.9	47	NR	NR	Glucomannan 3000 mg/day	Placebo
Li et al. (2022)	China	Parallel, R, PC, DB	Obese	69 B	38	31	5	31.14	30.60	31.41	31.22	Konjac flour NR	Lotus root starch (Placebo)
Fernandes et al. (2023)	Brazil	Parallel, R, PC, DB	Overweight individuals	42 F	21	21	2	28	30	29.41	32.39	Gummy candy enriched with Konjac glucomannan 500 mg/day	Placebo
Jenko Pražnikar et al. (2023)	Slovenia	Parallel, R, C	Overweight or obese participants	32 B	17	15	8	48.8	50.7	29.1	28	Glucomannan 3000 mg/day	Placebo

Abbreviations: B, both sexes; CG, control group; CO, controlled; DB, double‐blinded; F, Female; IG, intervention group; M, Male; NR, not reported; PC, placebo‐controlled; RA, randomized; SB, single‐blinded; T2D, Type 2 diabetes.

### 3.3 Risk of Bias Assessment

Risk of bias assessment that was performed according to the ROB 2 tool considered the general risk of bias as high for three included trials (Chearskul et al. 2007; Jenko Pražnikar et al. 2023; Walsh et al. 1984), and as low as in one eligible trial (Li et al. 2022). However, the levels of general risk of bias for the rest of the eligible studies were identified as having some concerns (Vuksan et al. 1999, 2000; Chen et al. 2003; Yoshida et al. 2006; Wood et al. 2007; Kaats et al. 2015; Fernandes et al. 2023). Details of the risk of bias assessment in each domain are exhibited in Figure 2.

**FIGURE 2:**
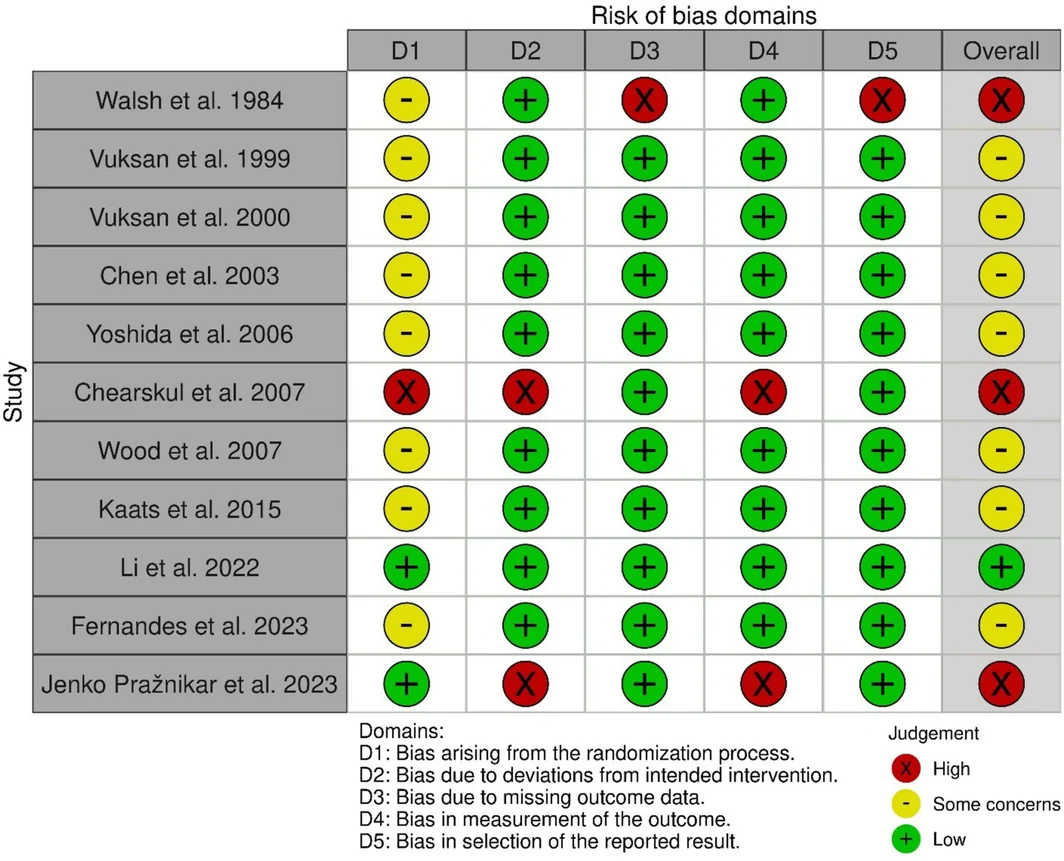
Risk of bias assessment plot.

### 3.4 Meta‐Analysis

#### 3.4.1 Impact of Glucomannan on Obesity Indices

##### 3.4.1.1 Impact of Glucomannan on Body Weight

Pooling nine effect sizes showed that glucomannan had no significant effect on body weight (WMD: 0.45 kg; 95% CI: −0.71 to 1.61; *p* = 0.44; Figure 3A). In addition, no significant heterogeneity was detected between the included trials (*I* ^2^ = 28.6%, and *p* = 0.19). Furthermore, in any of the pre‐defined subgroups, significant weight changes were not observed following glucomannan intake (Table S2).

**FIGURE 3:**
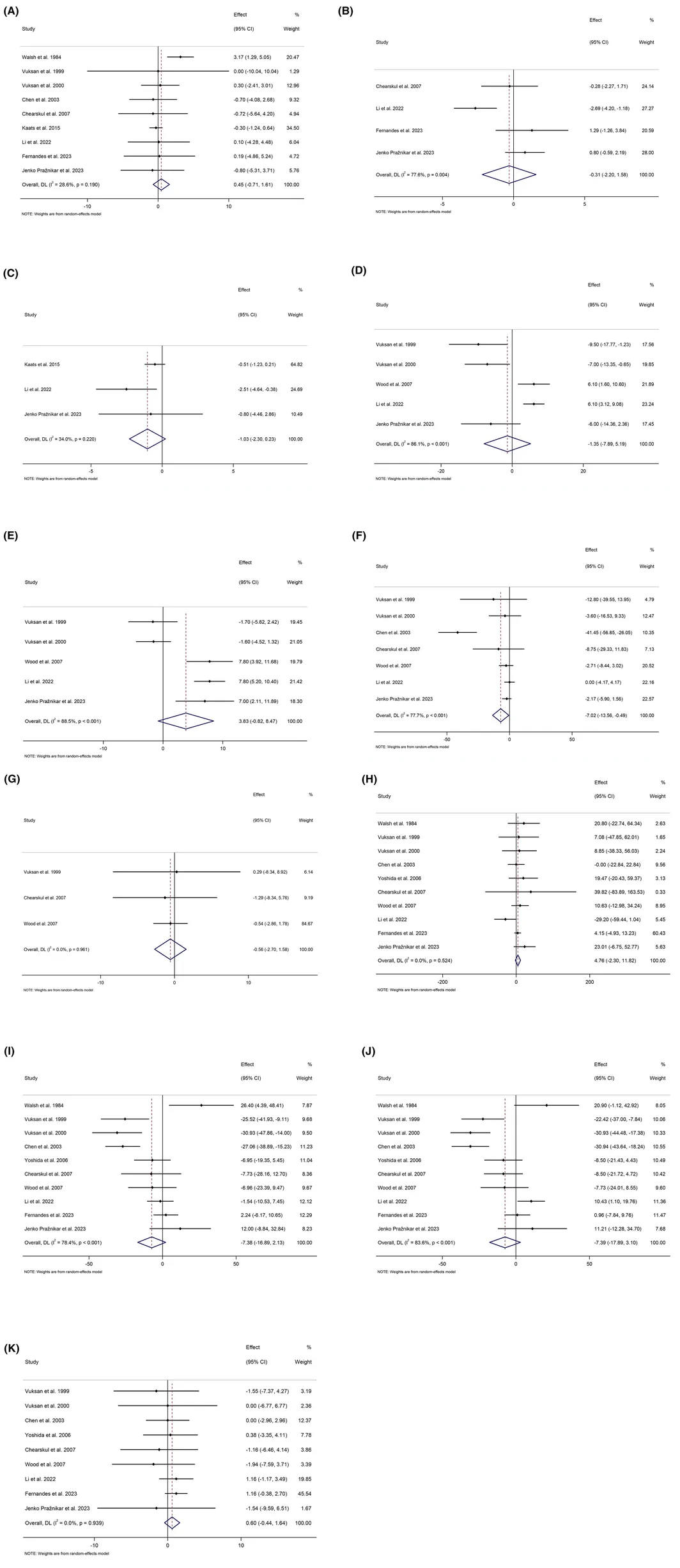
Forest plot detailing weighted mean difference and 95% confidence intervals (CIs) for the effect of Glucomannan on (A) body weight (kg); (B) BMI (kg/m^2^); (C) BFP (%); (D) SBP (mmHg); (E) DBP (mmHg); (F) FBG (mg/dL); (G) insulin (uU/mL); (H) TG (mg/dL); (I) TC (mg/dL); (J) LDL‐C (mg/dL); and (K) HDL‐C (mg/dL).

##### 3.4.1.2 Impact of Glucomannan on BMI

Combining four effect sizes showed that glucomannan did not lead to a significant change in BMI, overall or in any predefined criteria (WMD: −0.31 kg/m^2^; 95% CI: −2.20 to 1.58; *p* = 0.75) (Figure 3B; Table S2). However, there was significant heterogeneity among the included studies (*I* ^2^ = 77.6%, and *p* = 0.004).

##### 3.4.1.3 Impact of Glucomannan on BFP

Meta‐analyzing three effect sizes demonstrated that glucomannan had no significant impact on BFP (WMD: −1.03%; 95% CI: −2.30 to 0.23; *p* = 0.11; Figure 3C; Table S2). Also, no significant heterogeneity was detected between the included trials (*I* ^2^ = 34.0%, and *p* = 0.22).

#### 3.4.2 Impact of Glucomannan on Blood Pressure

##### 3.4.2.1 Impact of Glucomannan on SBP

Meta‐analysis of five effect sizes demonstrated that glucomannan had no significant effects on SBP (WMD: −1.35 mmHg; 95% CI: −7.89 to 5.19; *p* = 0.69; Figure 3D). Furthermore, significant heterogeneity was observed across the combined effect sizes (*I* ^2^ = 86.1%, and *p* < 0.001). Subgroup analysis revealed that glucomannan intake led to a significant reduction in SBP levels in the trials performed in Canada, those with a crossover design, individuals aged older than 50, participants with T2D, or studies that used glucomannan at a dosage higher than 3000 mg/day or for a duration of 4 weeks or shorter. In addition, glucomannan intake statistically significantly increased SBP levels in trials carried out in the USA; studies were conducted only in males or involved participants with mean BMI higher than 30 kg/m^2^ (Table S2).

##### 3.4.2.2 Impact of Glucomannan on DBP

Pooling five effect sizes showed that receiving glucomannan had no significant influence on DBP levels in comparison to the control groups (WMD: 3.83 mmHg; 95% CI: −0.81 to 8.47; *p* = 0.11; Figure 3E). In addition, significant heterogeneity was detected between included effect sizes (*I* ^2^ = 88.5%, and *p* < 0.001). Subgroup analyses demonstrated that glucomannan intake significantly increased DBP levels in studies that involved only males, as well as trials with a parallel design, participants aged ≤ 50, individuals with overweight/obesity, studies that were conducted in the USA, or consuming glucomannan with a dosage of ≤ 3000 mg/day or a duration longer than 4 weeks, as well as trials in populations with mean BMI higher than 30 kg/m^2^ (Table S2).

#### 3.4.3 Impact of Glucomannan on Glycemic Control

##### 3.4.3.1 Impact of Glucomannan on FBG

Pooling seven effect sizes revealed a significant reduction in FBG, followed by glucomannan intake (WMD: −7.02 mg/dL; 95% CI: −13.56 to −0.49; *p* = 0.04; Figure 3F). However, significant heterogeneity was detected between the included effect sizes (*I* ^2^ = 77.7%, and *p* < 0.001). Subgroup analyses to identify the source of heterogeneity among the eligible trials revealed that glucomannan intake significantly decreased FBG levels in trials conducted in individuals with mean BMI ranges of 25–29.9 kg/m^2^ and in studies involving both sexes. However, no significant changes in FBG levels were observed in the other pre‐defined subgroups (Table S2).

##### 3.4.3.2 Impact of Glucomannan on Fasting Insulin

Combining three effect sizes showed that glucomannan intake had no significant impact on fasting insulin levels compared with the control groups (WMD: −0.56 mU/mL; 95% CI: −2.70 to 1.58; *p* = 0.61; Figure 3G). Also, there was no significant heterogeneity among the pooled effect sizes (*I* ^2^ = 0.0%, and *p* = 0.96).

#### 3.4.4 Impact of Glucomannan on Lipid Profile

##### 3.4.4.1 Impact of Glucomannan on TG

Pooling ten effect sizes showed that glucomannan led to no significant change in TG levels overall or in any of the predefined criteria (WMD: 4.76 mg/dL; 95% CI: −2.30 to 11.82; *p* = 0.19; Figure 3H; Table 2). Also, there was no significant heterogeneity among the eligible trials (*I* ^2^ = 0.0%, and *p* = 0.52).

##### 3.4.4.2 Impact of Glucomannan on TC

Combining ten effect sizes demonstrated that glucomannan had no significant effect on TC levels (WMD: −7.38 mg/dL; 95% CI: −16.89 to 2.13; *p* = 0.13; Figure 3I; Table 2). However, significant heterogeneity was detected between the included effect sizes (*I* ^2^ = 78.4%, and *p* < 0.001). Subgroup analysis showed that glucomannan significantly reduced TC in trials that involved both sexes, included participants older than 50, were conducted in Canada, employed a cross‐over design, lasted up to 4 weeks, or used a glucomannan dosage above 3000 mg/day (Table 2).

##### 3.4.4.3 Impact of Glucomannan on LDL‐C

Meta‐analyzing ten effect sizes showed that glucomannan intake could not significantly alter LDL‐C levels (WMD: −7.39 mg/dL; 95% CI: −17.89 to 3.10; *p* = 0.17; Figure 3J). In addition, significant heterogeneity was observed between the included studies (*I* ^2^ = 83.6%, and *p* < 0.001). Subgroup analysis revealed that glucomannan significantly reduced LDL‐C in studies based in Canada, included both genders, performed on individuals older than 50, used a cross‐over design, had an intervention duration ≤ 4 weeks, administered more than 3000 mg/day of glucomannan, or were conducted on populations with mean BMI ranges of 25–29.9 kg/m^2^ (Table 2).

##### 3.4.4.4 Impact of Glucomannan on HDL‐C

Merging nine effect sizes demonstrated that glucomannan consumption had no significant effect on HDL‐C overall (WMD: 0.60 mg/dL; 95% CI: −0.44 to 1.64; *p* = 0.26; Figure 3K) (Table S2), or in any of the subgroups. Also, no significant heterogeneity was observed among the combined effect sizes (*I* ^2^ = 0.0%, and *p* = 0.94).

### 3.5 Linear and Non‐Linear Relationship

Meta‐regression demonstrated no significant linear relationship between intervention characteristics (dosage and duration of glucomannan consumption) and changes in body weight, TC, LDL‐C, HDL‐C, and TG levels (*p* > 0.05; Figure 4A–E presented linear dose–response relationships and Figure 5A–E presented linear duration–response relationships). Fractional polynomial modeling showed no significant non‐linear relationship between the dosage of glucomannan consumption and body weight, TG, TC, LDL‐C, and HDL‐C level changes (*p* > 0.05; Figure 6A–E). Also, the duration of consumption had no significant non‐linear association with changes in body weight, TG, LDL‐C, and HDL‐C levels (*p* > 0.05). However, a significant non‐linear relationship was found between the change in TC levels (*p* = 0.046) and the duration of glucomannan consumption (Figure 7A–E).

**FIGURE 4:**
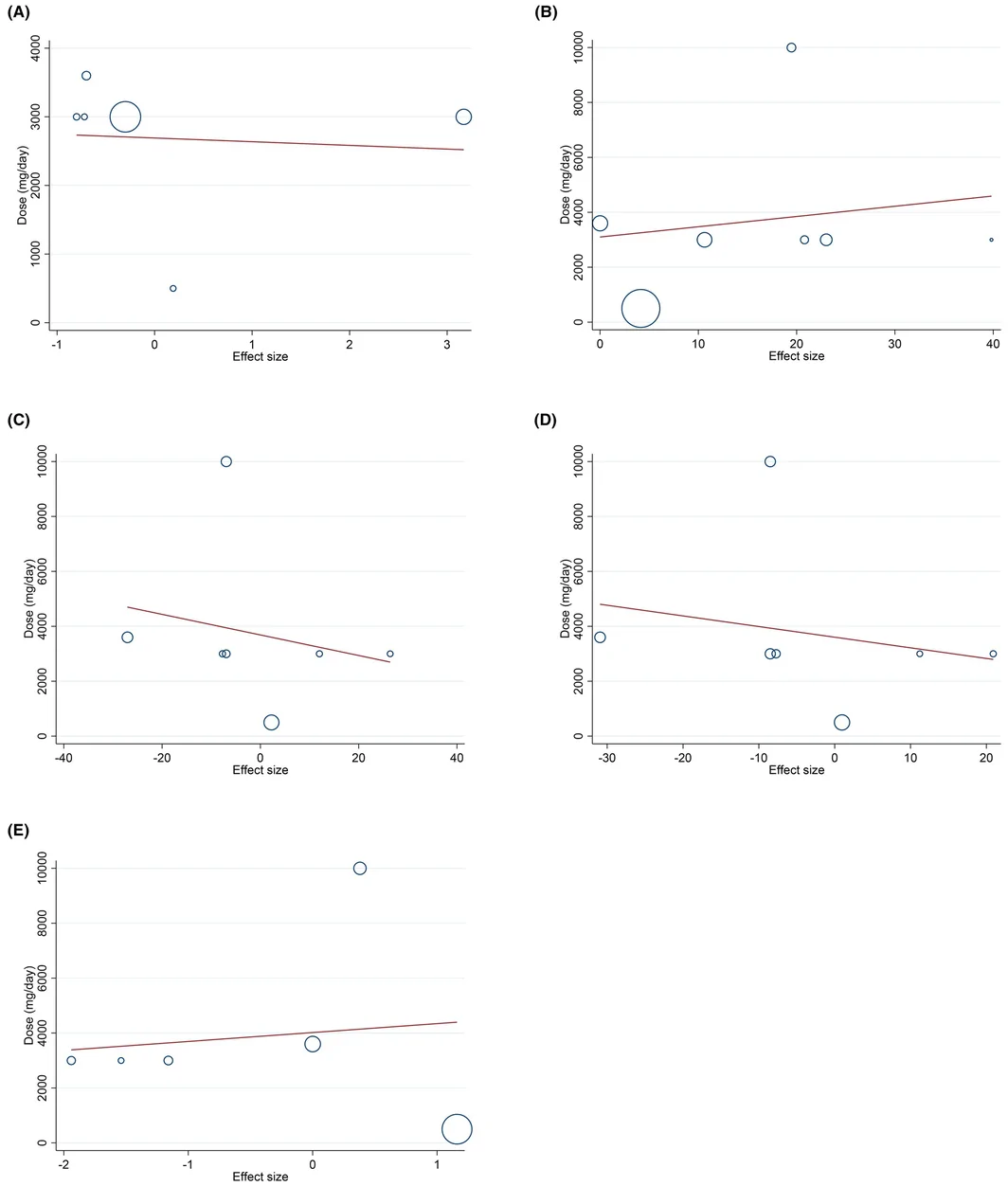
Linear relationships between glucomannan dosage (mg/day) and changes in (A) weight (kg), (B) TG (mg/dL), (C) TC (mg/dL), (D) LDL‐C (mg/dL), and (E) HDL‐C (mg/dL).

**FIGURE 5:**
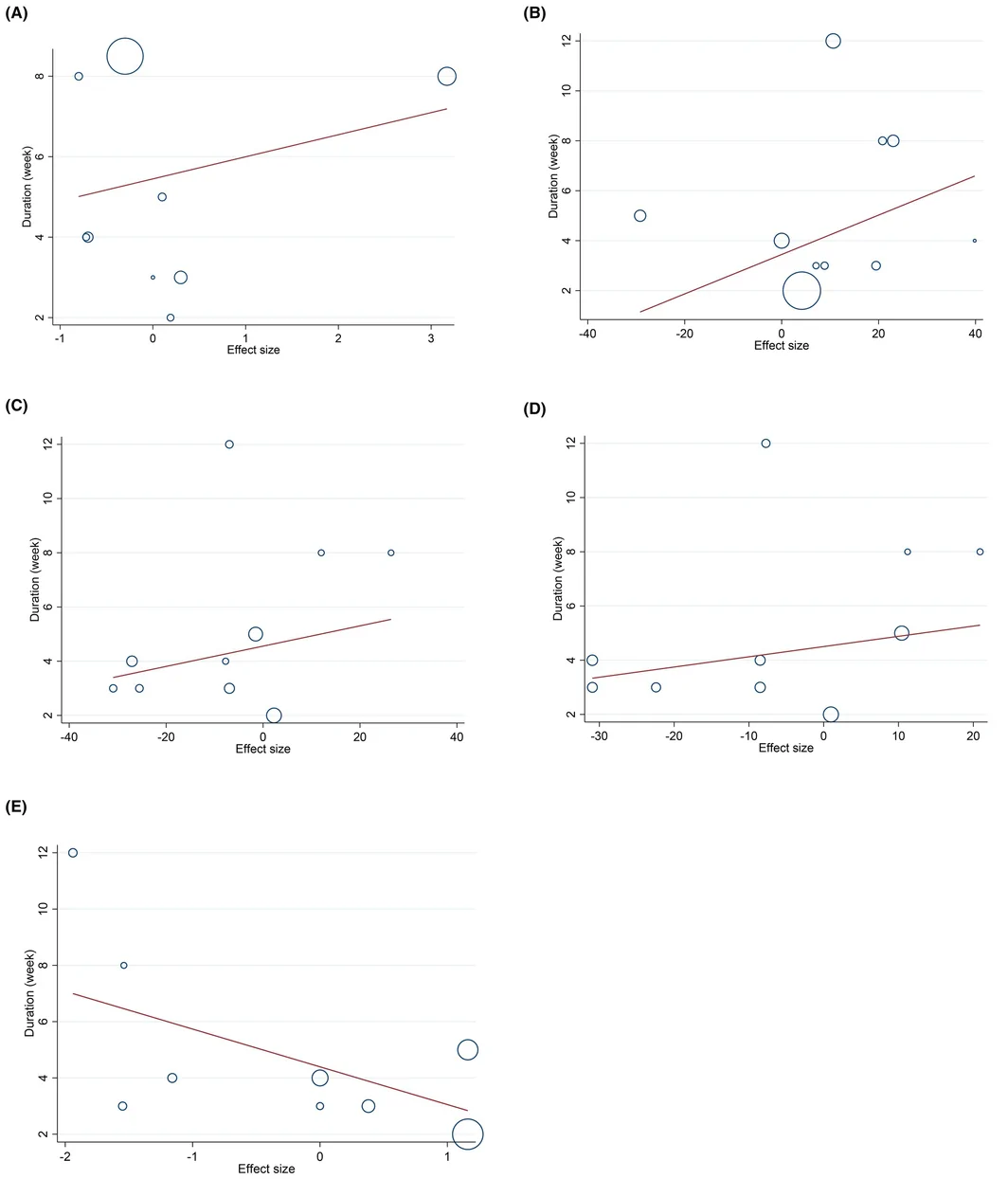
Linear relationships between glucomannan intervention duration (weeks) and changes in (A) weight (kg), (B) TG (mg/dL), (C) TC (mg/dL), (D) LDL‐C (mg/dL), and (E) HDL‐C (mg/dL).

**FIGURE 6:**
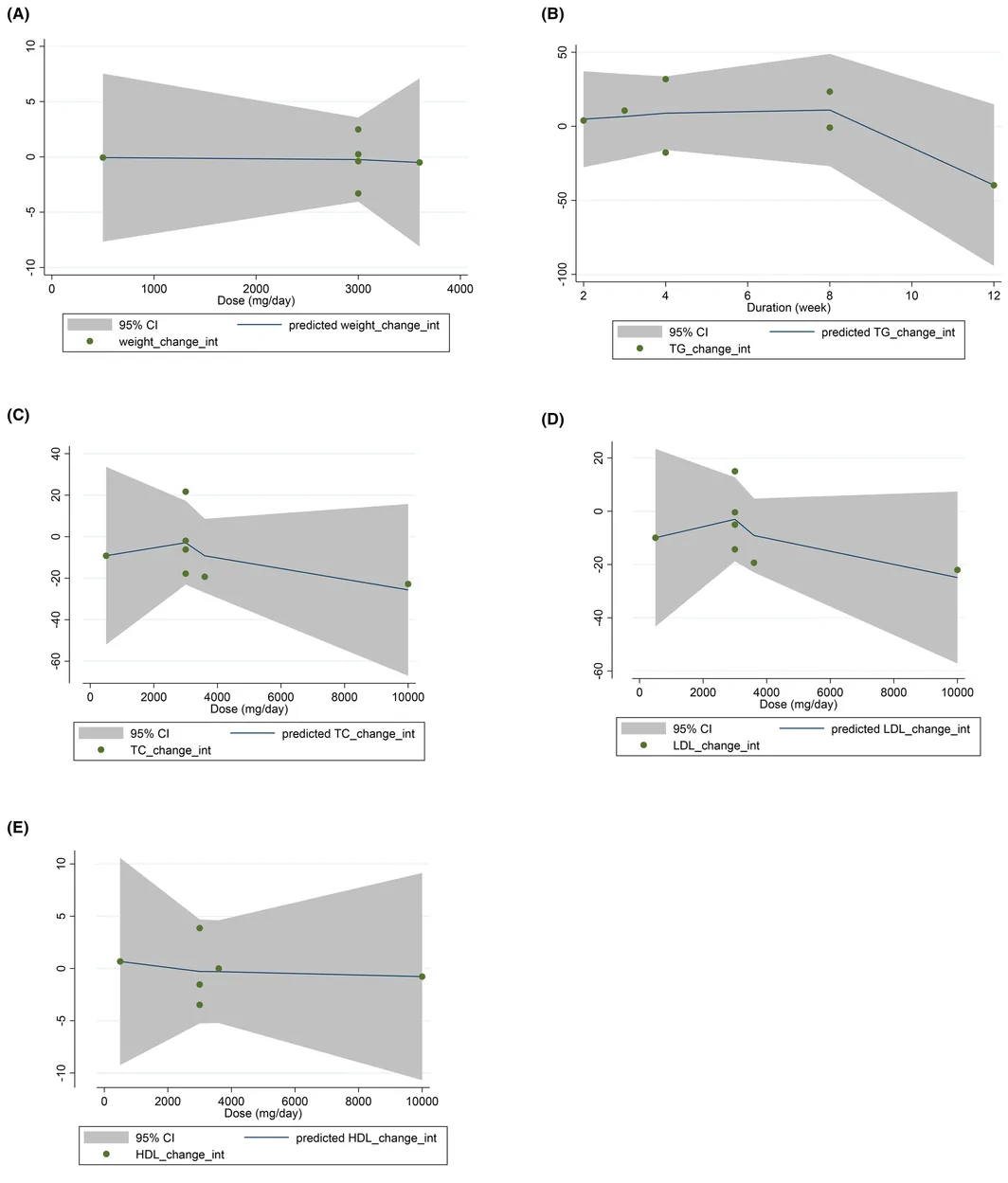
Non‐linear relationships between Glucomannan dosage intake (mg/day) and changes in (A) weight (kg), (B) TG (mg/dL), (C) TC (mg/dL), (D) LDL‐C (mg/dL), and (E) HDL‐C (mg/dL).

**FIGURE 7:**
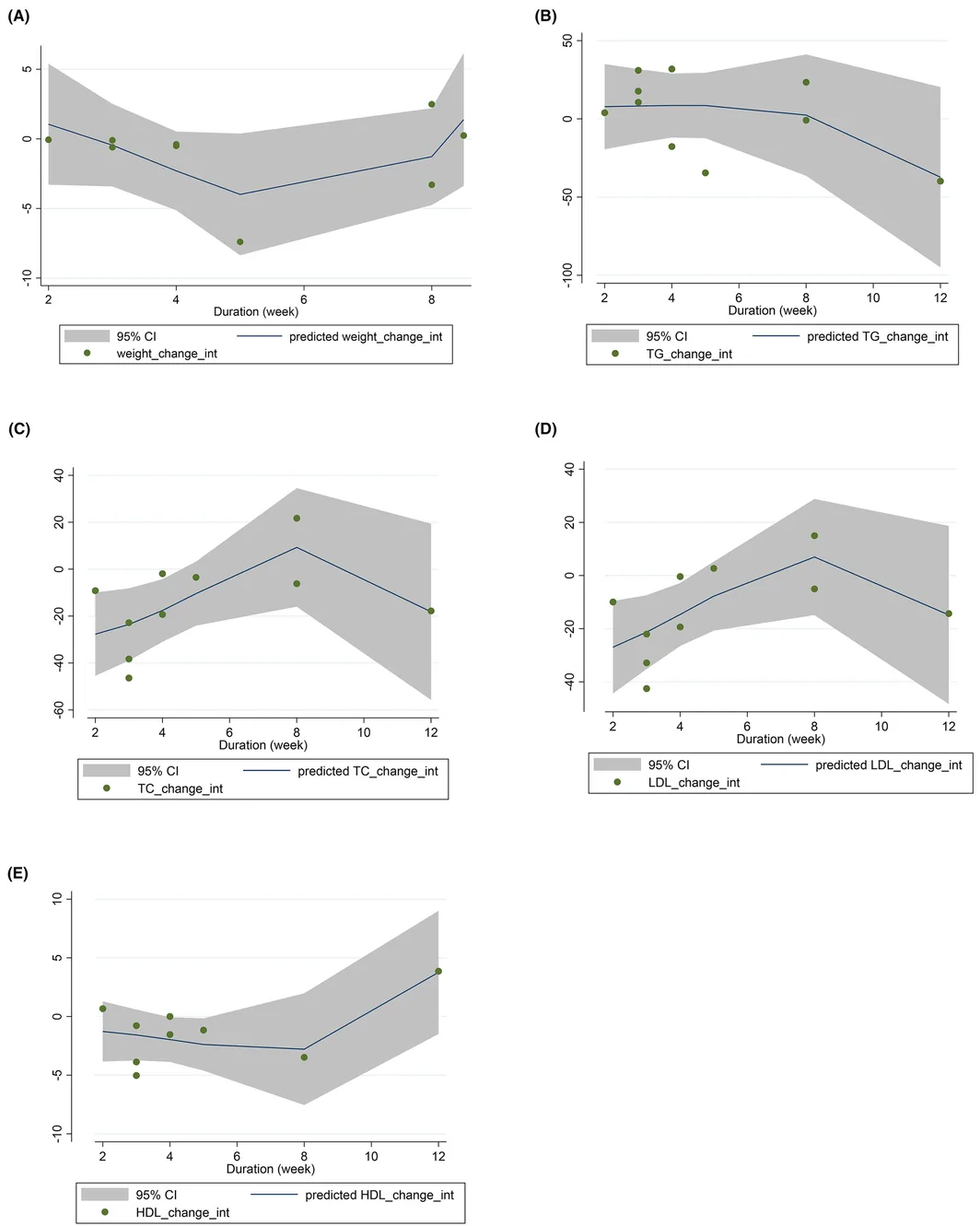
Non‐linear relationships between glucomannan intervention duration (weeks) and changes in (A) weight (kg), (B) TG (mg/dL), (C) TC (mg/dL), (D) LDL‐C (mg/dL), and (E) HDL‐C (mg/dL).

### 3.6 Sensitivity Analysis

#### 3.6.1 Sensitivity Analysis Based on Leave‐One‐Out Approach

Sensitivity analysis demonstrated significant changes in the overall effect size for FBG levels after omitting the effect size of Chen et al. (2003) (WMD: −1.76 mg/dL; 95% CI: −4.19 to 0.67; Chen et al. 2003). Also, the pooled effect size of DBP significantly changed after excluding the effect size of Vuksan et al. (1999) (WMD: 5.16 mmHg; 95% CI: 0.07, 10.25; Vuksan et al. 1999), or Vuksan et al. (2000) (WMD: 5.30 mmHg; 95% CI: 0.92 to 9.68; Vuksan et al. 2000). Furthermore, the overall effect sizes for TC levels changed significantly after removing the effect size of Walsh et al. (1984) (WMD: −10.23 mg/dL; 95% CI: −19.31, −1.15; Walsh et al. 1984). However, the pooled effect sizes for other outcomes did not significantly change following the omission of each included effect size.

#### 3.6.2 Sensitivity Analysis Assuming a Correlation Coefficient (R) of 0.5 for Crossover Trials

When a correlation coefficient (R) of 0.5 was assumed for crossover trials, the statistical significance of the pooled effects for FBG (WMD: −3.53 mg/dL; 95% CI: −8.35 to 1.29; *p* = 0.15), and DBP (WMD: 4.20 mmHg; 95% CI: 0.07 to 8.32; *p* = 0.046) changed compared with the primary analysis. However, for the other outcomes, the statistical significance of the pooled effect estimates remained consistent with that observed in the primary analysis (Table S3).

### 3.7 Publication Bias

Egger's regression test and visual inspection of the funnel plot detected significant publication bias in effect sizes investigating the impacts of glucomannan on SBP (*P*_Egger_ = 0.03), and HDL‐C levels (*P*_Egger_ = 0.001). However, no significant publication bias was detected in other assessed outcomes (Figure S1A–K).

### 3.8 GRADE Analysis

The GRADE analysis identified the quality of evidence for the impacts of glucomannan intake on weight, BFP, and TG levels as moderate. Additionally, the quality of evidence was downgraded to low for fasting insulin, FBG, and HDL‐C levels, and to very low for BMI, SBP, DBP, TC, and LDL‐C levels (Table S4).

## 4 Discussion

In the present meta‐analysis, we evaluated the effects of glucomannan on cardiometabolic parameters in adults with insulin resistance states. Overall, we found that glucomannan significantly reduced FBG levels; however, this finding was not statistically significant under the alternative correlation assumption for cross‐over studies, suggesting that the FBG effect may be sensitive to the assumptions used in the analysis. In addition, glucomannan consumption did not significantly affect insulin levels, blood pressure, obesity indices (body weight, BMI, BFP), or lipid profile (TG, TC, LDL‐C, and HDL‐C). However, using the alternative correlation assumption for cross‐over studies changed the significance of FBG.

Subgroup analyses revealed that higher doses (> 3000 mg/day) and shorter intervention durations (≤ 4 weeks) were associated with significant reductions in TC and LDL‐ *C. meta* ‐regression showed no significant linear relationship between dosage or duration of glucomannan supplementation and changes in body weight or TC, TG, LDL‐C, and HDL‐C levels. Also, no significant non‐linear association was detected between glucomannan dosage and changes in these outcomes. Furthermore, there was no significant non‐linear relationship between durations of glucomannan intake and body weight, TG, LDL‐C, and HDL‐C level changes. However, a significant non‐linear relationship was observed between changes in TC levels and the duration of glucomannan intake. These findings suggest that while glucomannan may be a useful adjunct for improving glycemic control in insulin‐resistant individuals, its broader cardiometabolic benefits appear to be context‐dependent and inconsistent. However, due to the small number of effect sizes in some subgroups, the findings of subgroup analyses should be considered exploratory and interpreted with caution.

In the present study, significant heterogeneity was detected among the pooled effect sizes for BMI, FBG, SBP, DBP, TC, and LDL‐C. This heterogeneity may be attributed to the clinical and methodological differences across the included studies. Subgroup analyses showed that the significant heterogeneity may be explained by differences in the country of origin of the study, study design, intervention dosage and duration, and features of participants, including the mean age and health status.

Our results align with prior meta‐analyses showing the potential FBG‐lowering effect of glucomannan in T2D populations (Zhang et al. 2023). Zhang et al. (2023) found that glucomannan supplementation significantly reduced glycemic markers (fasting and postprandial glucose, insulin, and fructosamine) and improved lipid profiles (TC and LDL‐C) in individuals with T2D. These results partially align with our findings for FBG‐lowering effects of glucomannan; however, they contrast with our non‐significant results for other glycemic and lipid parameters. These inconsistencies may be due to differences in study populations, as Zhang et al.'s meta‐analysis focused exclusively on individuals with T2D. In contrast, our analysis included a broader range of insulin resistance states. While both analyses support glucomannan's hypoglycemic properties, the more comprehensive metabolic benefits observed in Zhang et al.'s study suggest that its effects may be more pronounced in patients with T2D. The FPG‐lowering effects of glucomannan, in particular, may be attributed to its high viscosity, which slows gastric emptying and delays carbohydrate absorption (Vuksan et al. 2000). Furthermore, our findings regarding weight reduction are consistent with those of a study conducted by Onakpoya et al. (2014) that included studies involving individuals with overweight and obesity, which found no statistically significant effect of glucomannan on body weight, based on pooled results from eight effect sizes (MD: −0.22 kg; 95% CI: −0.62, 0.19). Sood et al. found that glucomannan significantly improved several cardiometabolic markers, including TC, LDL‐C, TG, body weight, and FBG, but had no significant effect on HDL‐C or blood pressure. This study indicated smaller benefits in pediatric populations, individuals undergoing dietary modifications, or those with impaired glucose metabolism (Sood et al. 2008). Our meta‐analysis similarly confirms FBG‐lowering effects of glucomannan consumption but did not demonstrate significant changes in other cardiometabolic outcomes. These discrepancies may reflect differences in the inclusion criteria of our studies.

Another meta‐analysis conducted by Musazadeh et al. (2024) showed that glucomannan significantly reduced TC and LDL‐C levels in adults without affecting TG, HDL‐C, LDL‐C/HDL‐C ratio, Apo A1, or APO‐B/A1 ratio. These results were inconsistent with our overall findings, which indicated no significant effect of glucomannan intake on lipid profiles. These divergent findings may stem from the lack of limitation of inclusion criteria in this review to exclusively populations with an insulin resistance status. Also, these inconsistencies underscore the need to consider population‐specific differences when interpreting the efficacy of glucomannan supplementation. Subgroup analyses suggested greater improvements in TC and LDL‐C levels among studies with shorter intervention durations (≤ 4 weeks) or higher dosages (> 3000 mg/day). However, these findings should be considered exploratory and interpreted as hypothesis‐generating rather than as recommendations regarding a specific dose or intervention duration.

The existing evidence suggests glucomannan's effects on body weight, BMI, glycemic control, lipid profiles, and blood pressure are thought to arise from its highly viscous, soluble fiber properties, which slow glucose absorption and enhance short‐chain fatty acid production via gut microbiota fermentation (Qi et al. 2022; Mirzababaei et al. 2022). In addition, as a highly viscous soluble fiber, it may interfere with intestinal absorption of both dietary cholesterol and bile acids through physical entrapment, increasing fecal excretion (Musazadeh et al. 2024). Also, its gel‐forming properties delay gastric emptying, which may alter nutrient absorption kinetics and contribute to improved lipid handling (Zhang et al. 2020; Xu et al. 2023).

Our study has several strengths. Firstly, it is the first meta‐analysis to comprehensively investigate the effects of glucomannan across various insulin resistance states, not just T2D or obesity. Secondly, we performed a comprehensive subgroup analysis to identify possible sources of heterogeneity and to evaluate the effects of glucomannan intake in different pre‐defined subgroups. Third, we performed meta‐regression and dose–response analyses to assess linear and non‐linear relationships between the features of intervention and changes in lipid profile and weight, respectively. Finally, we used the Cochrane RoB 2 tool, the most up‐to‐date tool for assessing risk of bias.

However, our study has some limitations. A limited number of trials were included in this review. In addition, there was significant heterogeneity across the included effect sizes for some outcomes, such as BMI, FBG, SBP, DBP, TC, and LDL‐C. Also, the levels of evidence quality for several outcomes, including BMI, fasting insulin, FBG, SBP, DBP, TC, LDL‐C, and HDL‐C, ranged from low to very low. Potential publication bias was also detected for SBP and HDL‐C. Also, the results of the publication bias investigation for obesity indices, glycemic markers, and blood pressure need to be interpreted cautiously due to the limited number of included studies (Cumpston et al. 2019). In addition, sensitivity analyses showed that pooled results for FBG, TC, and DBP were influenced by single studies, indicating instability in some effect estimates. Furthermore, only one included trial was identified as having a low overall risk of bias. In addition, due to the small number of studies, the findings of meta‐regression and dose–response analysis should be interpreted cautiously and were not used for exploring the optimum supplementation features, including dosage and duration.

## 5 Conclusion

The findings of this meta‐analysis suggest that glucomannan supplementation may have a potential beneficial effect on fasting blood glucose in adults with insulin resistance‐related conditions. However, this finding should be interpreted with caution given the substantial heterogeneity, the sensitivity of the FBG result to the removal of one study, the low certainty of the available evidence, and the sensitivity of the effect estimate to the assumed correlation coefficient. When an alternative correlation assumption was applied, the statistically significant effect of glucomannan supplementation on FBG was no longer observed. In addition, our review showed that glucomannan supplementation may have had no significant effect on blood pressure or lipid profile in these participants. Future well‐designed, large‐scale RCTs are needed to clarify the optimal dosage, duration, and patient populations most likely to benefit, and to further investigate the underlying mechanisms of action.

## Author Contributions

**Mostafa Shahraki Jazinaki:** conceptualization, investigation, writing – original draft, formal analysis. **Hossein Rafiei:** writing – original draft, writing – review and editing. **Hossein Bahari:** writing – original draft, writing – review and editing, project administration. **Naseh Pahlavani:** validation, methodology, supervision, writing – original draft.

## Funding

The authors have nothing to report.

## Conflicts of Interest

The authors declare no conflicts of interest.

## Supporting information


**Table S1:** Details of the search strategy applied in each database.
**Table S2:** Subgroup analyses of Glucomannan intake on obesity indicators, blood pressure, glycemic control, and lipid profile.
**Table S3:** Sensitivity analysis of the pooled effect sizes using an alternative within‐participant correlation coefficient (R = 0.5) for cross‐over trials and (R = 0.8) for parallel trials.
**Table S4:** GRADE profile of glucomannan consumption for cardiometabolic parameters.
**Figure S1:** Funnel plots for the effect of Glucomannan on (A) body weight (kg); (B) BMI (kg/m2); (C) BFP (%); (D) SBP (mmHg); (E) DBP (mmHg), (F) FBG (mg/dL), (G) insulin (uU/mL), (H) TG (mg/dL), (I) TC (mg/dL), (J) LDL‐C (mg/dL), and (K) HDL‐C (mg/dL).

## Data Availability

All data generated or analyzed during this study are included in this published article.
