# Supplementary material for: Effects of Glucomannan on Cardiometabolic Parameters in Adults With Insulin Resistance‐Related Conditions: A Systematic Review and Dose–Response Meta‐Analysis
Source: Food Sci Nutr. 2026 Oct 8;14(10):e72460. doi: 10.1002/fsn3.72460 (PMC13650263; doi:10.1002/fsn3.72460)
Supplement: Supplementary file 1 [file FSN3-14-e72460-s001.docx]

**Effects of Glucomannan on Cardiometabolic Parameters in Adults with Insulin Resistance-related Conditions: A Systematic Review and Dose-Response Meta-analysis**

Electronic Supplementary Material: Appendix Tables S1-S4, and Figure S1

**Supplementary Table 1.** Details of the search strategy applied in each database

Search date: March 2025

| **PubMed** | (("Glucomannan"[Title/Abstract]) OR ("Amorphophallus konjac"[Title/Abstract]) OR ("konjac"[Title/Abstract]) OR ("konnyaku"[Title/Abstract]) OR (devils tongue[Title/Abstract]) OR ("elephant foot"[Title/Abstract])) AND (("Diabetes"[Title/Abstract]) OR ("PCOS"[Title/Abstract]) OR ("Polycystic ovary syndrome"[Title/Abstract]) OR ("metabolic syndrome"[Title/Abstract]) OR ("Syndrome X"[Title/Abstract]) OR ("Insulin resistance syndrome"[Title/Abstract]) OR ("Obese"[Title/Abstract]) OR ("obesity"[Title/Abstract]) OR ("Overweight"[Title/Abstract]) OR ("Insulin resistance"[Title/Abstract]) OR ("Prediabetes"[Title/Abstract]) OR ("Hyperinsulinemia"[Title/Abstract]) OR ("Glucose intolerance"[Title/Abstract]) OR ("hyperglycemia"[Title/Abstract]) OR ("Diabetes Mellitus"[Mesh]) OR "Obesity"[Mesh] OR "Overweight"[Mesh] OR "Metabolic Syndrome"[Mesh]) AND (("randomised"[Title/Abstract]) OR ("random"[Title/Abstract]) OR ("randomly"[Title/Abstract]) OR ("placebo"[Title/Abstract]) OR ("clinical trial"[Title/Abstract]) OR ("Trial"[Title/Abstract]) OR ("randomized controlled trial"[Title/Abstract]) OR ("randomized clinical trial"[Title/Abstract]) OR (RCT[Title/Abstract]) OR ("blinded"[Title/Abstract]) OR ("double blind"[Title/Abstract]) OR ("double blinded"[Title/Abstract]) OR ("clinical trial"[Title/Abstract]) OR ("Pragmatic Clinical Trial"[Title/Abstract]) OR ("Cross-Over"[Title/Abstract]) OR ("parallel"[Title/Abstract]) OR ("randomized controlled trial"[MeSH Terms]) OR ("Intervention"[Title/Abstract]) OR ("Intervention Study"[Title/Abstract]) OR ("Intervention Studies"[Title/Abstract]) OR ("controlled trial"[Title/Abstract]) OR ("randomized"[Title/Abstract])) |
| --- | --- |
| **Scopus** | ( ( ( TITLE-ABS-KEY ( "Glucomannan" ) OR TITLE-ABS-KEY ( "Amorphophallus konjac" ) OR TITLE-ABS-KEY ( "konjac" ) OR TITLE-ABS-KEY ( "konnyaku" ) OR TITLE-ABS-KEY ( "devils tongue" ) OR TITLE-ABS-KEY ( "elephant foot" ) ) ) AND ( ( TITLE-ABS-KEY ( "Intervention" ) OR TITLE-ABS-KEY ( "Intervention Study" ) OR TITLE-ABS-KEY ( "Intervention Studies" ) OR TITLE-ABS-KEY ( "controlled trial" ) OR TITLE-ABS-KEY ( "randomized" ) OR TITLE-ABS-KEY ( "randomised" ) OR TITLE-ABS-KEY ( "random" ) OR TITLE-ABS-KEY ( "random" ) OR TITLE-ABS-KEY ( "randomly" ) OR TITLE-ABS-KEY ( "placebo" ) OR TITLE-ABS-KEY ( "clinical trial" ) OR TITLE-ABS-KEY ( "Trial" ) OR TITLE-ABS-KEY ( "randomized controlled trial" ) OR TITLE-ABS-KEY ( "randomized clinical trial" ) OR TITLE-ABS-KEY ( "RCT" ) OR TITLE-ABS-KEY ( "blinded" ) OR TITLE-ABS-KEY ( "double blind" ) OR TITLE-ABS-KEY ( "double blinded" ) OR TITLE-ABS-KEY ( "clinical trial" ) OR TITLE-ABS-KEY ( "Pragmatic Clinical Trial" ) OR TITLE-ABS-KEY ( "Cross-Over" ) OR TITLE-ABS-KEY ( "parallel" ) ) ) ) AND ( ( TITLE-ABS-KEY ( "Diabetes" ) OR TITLE-ABS-KEY ( "PCOS" ) OR TITLE-ABS-KEY ( "Polycystic ovary syndrome" ) OR TITLE-ABS-KEY ( "metabolic syndrome" ) OR TITLE-ABS-KEY ( "Syndrome X" ) OR TITLE-ABS-KEY ( "Insulin resistance syndrome" ) OR TITLE-ABS-KEY ( "Obese" ) OR TITLE-ABS-KEY ( "obesity" ) OR TITLE-ABS-KEY ( "Overweight" ) OR TITLE-ABS-KEY ( "Insulin resistance" ) OR TITLE-ABS-KEY ( "Prediabetes" ) OR TITLE-ABS-KEY ( "Hyperinsulinemia" ) OR TITLE-ABS-KEY ( "Glucose intolerance" ) OR TITLE-ABS-KEY ( "hyperglycemia" ) ) ) |
| **Web of Science** | **Query #1**  (((((((((((((TS=("Diabetes")) OR TS=("PCOS")) OR TS=("Polycystic ovary syndrome")) OR TS=("metabolic syndrome")) OR TS=("Syndrome X")) OR TS=("Insulin resistance syndrome")) OR TS=("Obese")) OR TS=("obesity")) OR TS=("Overweight")) OR TS=("Insulin resistance")) OR TS=("Prediabetes")) OR TS=("Hyperinsulinemia")) OR TS=("Glucose intolerance")) OR TS=("hyperglycemia")  **Query #2**  ((((((((((((((((((((TS=("Intervention")) OR TS=("Intervention Study")) OR TS=("Intervention Studies")) OR TS=("controlled trial")) OR TS=("randomized")) OR TS=("randomised")) OR TS=("random")) OR TS=("randomly")) OR TS=("placebo")) OR TS=("clinical trial")) OR TS=("Trial")) OR TS=("randomized controlled trial")) OR TS=("randomized clinical trial")) OR TS=("RCT")) OR TS=("blinded")) OR TS=("double blind")) OR TS=("double blinded")) OR TS=("clinical trial")) OR TS=("Pragmatic Clinical Trial")) OR TS=("Cross-Over")) OR TS=("parallel")  **Query #3**  (((((TS=("Glucomannan")) OR TS=("Amorphophallus konjac")) OR TS=("konjac")) OR TS=("konnyaku")) OR TS=("devils tongue")) OR TS=("elephant foot" )  **Final query: #1 AND #2 AND #3** |

**Supplementary Table** **2**. Subgroup analyses of Glucomannan intake on obesity indicators, blood pressure, glycemic control, and lipid profile.

|  | NO | WMD (95%CI) | P-value | heterogeneity | | |
| --- | --- | --- | --- | --- | --- | --- |
|  |  |  |  | P heterogeneity | I^2^ | P between sub-groups |
| Subgroup analyses of Glucomannan on weight (Kg) | | | | | |  |
| Overall effect | 9 | 0.45 (-0.71, 1.61) | 0.44 | 0.19 | 28.6% |  |
| Country | | | | | | |
| USA | 2 | 1.33 (-2.06, 4.73) | 0.44 | **0.001** | 90.4% | 0.66 |
| Canada | 2 | 0.28 (-2.34, 2.90) | 0.83 | 0.95 | 0.0% |  |
| Others | 6 | -0.44 (-2.36, 1.49) | 0.66 | 0.99< | 0.0% |  |
| Study design | | | | | | |
| Paralell | 5 | 0.72 (-1.23, 2.68) | 0.46 | **0.03** | 62.6% | 0.51 |
| Cross-over | 4 | -0.18 (-2.09, 1.72) | 0.85 | 0.97 | 0.0% |  |
| Gender | | | | | | |
| Both sexes | 7 | -0.28 (-1.10, 0.53) | 0.50 | 0.99< | 0.0% | **0.004** |
| Female | 1 | 0.19 (-4.86, 5.24) | 0.94 | - | - |  |
| NR | 1 | 3.17 (1.29, 5.06) | **0.001** | - | - |  |
| Age | | | | | | |
| ≤50 | 4 | -0.29 (1.18, 0.60) | 0.53 | 0.99 | 0.0% | **0.004** |
| >50 | 4 | 0.18 (-2.09, 1.72) | 0.85 | 0.97 | 0.0% |  |
| NR | 1 | 3.17 (1.26, 5.06) | **0.001** | - | - |  |
| Trial duration (weeks) | | | | | | |
| ≤4 | 5 | -0.14 (-1.92, 1.65) | 0.88 | 0.99 | 0.0% | 0.53 |
| >4 | 4 | 0.77 (-1.47, 3.02) | 0.50 | **0.01** | 72.0% |  |
| Intervention dose (mg/day) | | | | | | |
| ≤3000 | 5 | 0.64 (1.37, 2.64) | 0.53 | 0.03 | 63.2% | 0.88 |
| >3000 | 3 | -0.09 (-2.16, 1.98) | 0.93 | 0.90 | 0.0% |  |
| NR | 1 | 0.10 (4.29, 4.49) | 0.96 | - | - |  |
| Health status | | | | | | |
| T2DM | 4 | 1.11 (-1.45, 3.67) | 0.40 | 0.15 | 42.8% | 0.57 |
| Overweight/Obesity | 4 | -0.28 (-1.17, 0.59) | 0.52 | 0.99 | 0.0% |  |
| Others | 1 | 0.30 (-2.41, 3.01) | 0.83 | - | - |  |
| Baseline BMI (kg/m^2^) | | | | | | |
| 25-29.9 | 4 | -0.29 (-2.07, 1.50) | 0.75 | 0.96 | 0.0% | 0.71 |
| >30 | 2 | 0.14 (-3.17, 3.45) | 0.94 | 0.98 | 0.0% |  |
| NR | 3 | 1.22 (-1.83, 4.27) | 0.43 | **0.005** | 80.8% |  |
| Subgroup analyses of Glucomannan on BMI (Kg/m^2^) | | | | | | |
| Overall effect | 4 | -0.31 (-2.20, 1.58) | 0.75 | **0.004** | 77.6% |  |
| Study design | | | | | | |
| Paralell | 3 | -0.29 (-2.88, 2.31) | 0.83 | **0.001** | 85.0% | 0.99< |
| Cross-over | 1 | -0.28 (-2.27, 1.71) | 0.78 | - | - |  |
| Gender | | | | | | |
| Both sexes | 3 | -0.73 (-2.94, 1.49) | 0.52 | **0.003** | 82.3% | 0.24 |
| Female | 1 | 1.29 (-1.26, 3.84) | 0.32 | - | - |  |
| Age | | | | | | |
| ≤50 | 3 | -2.30 (-2.89, 2.31) | 0.83 | **0.001** | 85.0% | 0.99< |
| >50 | 1 | -0.28 (-2.27, 1.710 | 0.78 | - | - |  |
| Trial duration (weeks) | | | | | | |
| ≤4 | 2 | 0.32 (-1.27, 1.89) | 0.70 | 0.34 | 0.0% | 0.52 |
| >4 | 2 | -0.93 (-4.35, 2.49) | 0.59 | **0.001** | 91.0% |  |
| Intervention dose (mg/day) | | | | | | |
| ≤3000 | 3 | 0.59 (-0.46, 1.63) | 0.27 | 0.58 | 0.0% | **<0.001** |
| NR | 1 | -2.69 (-4.19, -1.18) | **<0.001** | - | - |  |
| Health status | | | | | | |
| T2DM | 1 | -0.28 (-2.27, 1.71) | 0.78 | - | - | 0.99< |
| Overweight/Obesity | 3 | -0.29 (-2.88, 2.31) | 0.83 | **0.001** | 85.0% |  |
| Baseline BMI (kg/m^2^) | | | | | | |
| Overweight (25-29.9) | 2 | 0.45 (-0.70, 1.59) | 0.44 | 0.38 | 0.0% | 0.53 |
| Obesity (>30) | 2 | -0.84 (-4.73, 3.05) | 0.67 | **0.009** | 85.5% |  |
| Subgroup analyses of Glucomannan on BFP (%) | | | | | | |
| Overall effect | 3 | -1.03 (-2.30, 0.23) | 0.11 | 0.22 | 34.0% |  |
| Subgroup analyses of Glucomannan on FBG (mg/dl) | | | | | | |
| Overall effect | 7 | -7.02 (-13.56, -0.49) | **0.04** | **<0.001** | 77.7% |  |
| Country | | | | | | |
| USA | 1 | -2.71 (-8.44, 3.02) | 0.35 | - | - | 0.47 |
| Canada | 2 | -5.34 (-16.99, 6.30) | 0.37 | 0.54 | 0.0% |  |
| Others | 4 | -10.17 (-20.62, 0.28) | 0.06 | **<0.001** | 88.6% |  |
| Study design | | | | | | |
| Paralell | 3 | -1.49 (-4.00, 1.01) | 0.24 | 0.67 | 0.0% | 0.12 |
| Cross-over | 4 | -16.98 (-36.73, 2.79) | 0.09 | **0.002** | 79.3% |  |
| Gender | | | | | | |
| Both sexes | 6 | -8.93 (-17.36, -0.49) | **0.04** | **<0.001** | 81.4% | 0.23 |
| Male | 1 | -2.71 (-8.44, 3.02) | 0.35 | - | - |  |
| Age | | | | | | |
| ≤50 | 3 | -1.49 (-3.99, 1.00) | 0.24 | 0.67 | 0.0% | 0.13 |
| >50 | 4 | -16.98 (-36.73, 2.79) | 0.09 | **0.002** | 79.3% |  |
| Trial duration (weeks) | | | | | | |
| ≤4 | 4 | -16.98 (-36.73, 2.79) | 0.09 | **0.002** | 79.3% | 0.13 |
| >4 | 3 | -1.49 (-3.99, 1.00) | 0.24 | 0.67 | 0.0% |  |
| Intervention dose (mg/day) | | | | | | |
| ≤3000 | 3 | -2.48 (-5.57, 0.61) | 0.12 | 0.82 | 0.0% | 0.27 |
| >3000 | 3 | -19.53 (-45.87, 6.82) | 0.15 | **0.001** | 85.5% |  |
| NR | 1 | 0.00 (-4.17, 4.17) | 0.99< | - | - |  |
| Health status | | | | | | |
| T2DM | 3 | -22.37 (-45.29, 0.57) | 0.06 | **0.02** | 73.3% | 0.20 |
| Overweight/Obesity | 3 | -1.49 (-4.00, 1.01) | 0.25 | 0.67 | 0.0% |  |
| Others | 1 | -3.60 (-16.52, 9.33) | 0.59 | - | - |  |
| Baseline BMI (kg/m^2^) | | | | | | |
| 25-29.9 | 5 | -9.81 (-19.41, -0.21) | **0.04** | **<0.001** | 83.4% | 0.13 |
| >30 | 1 | 0.00 (-4.17, 4.17) | 0.35 | - | - |  |
| NR | 1 | -12.80 (-39.55, 13.95) | 0.99< | - | - |  |
| Subgroup analyses of Glucomannan on Insulin (uU/mL) | | | | | | |
| Overall effect | 3 | -0.56 (-2.70, 1.58) | 0.61 | 0.96 | 0.0% |  |
| Subgroup analyses of Glucomannan on SBP (mmHg) | | | | | | |
| Overall effect | 5 | -1.35 (-7.89, 5.19) | 0.69 | **<0.001** | 86.1% |  |
| Country | | | | | | |
| USA | 1 | 6.10 (1.60, 10.60) | **0.008** | - | - | **<0.001** |
| Canada | 2 | -7.93 (-12.96, -2.89) | **0.002** | 0.64 | 0.0% |  |
| Others | 2 | 0.70 (-11.08, 12.49) | 0.90 | **0.008** | 86.0% |  |
| Study design | | | | | | |
| Paralell | 3 | 3.41 (-1.97, 8.79) | 0.21 | **0.03** | 73.0% | **0.003** |
| Cross-over | 2 | -7.93 (-12.96, -2.89) | **0.002** | 0.64 | 0.0% |  |
| Gender | | | | | | |
| Both sexes | 4 | -3.66 (-12.57, 5.26) | 0.42 | <0.001 | 88.4% | 0.06 |
| Male | 1 | 6.10 (1.60, 10.60) | **0.008** | - | - |  |
| Age | | | | | | |
| ≤50 | 3 | 3.41 (-1.96, 8.79) | 0.21 | **0.02** | 73.0% | **0.003** |
| >50 | 2 | -7.92 (-12.96, -2.89) | **0.002** | 0.63 | 0.0% |  |
| Trial duration (weeks) | | | | | | |
| ≤4 | 2 | -7.93 (-12.97, -2.90) | **0.002** | 0.64 | 0.0% | **0.003** |
| >4 | 3 | 3.41 (-1.97, 8.79) | 0.21 | **0.03** | 73.0% |  |
| Intervention dose (mg/day) | | | | | | |
| ≤3000 | 2 | 0.58 (-11.23, 12.40) | 0.92 | **0.01** | 84.0% | **<0.001** |
| >3000 | 2 | -7.93 (-12.96, -2.90) | **0.002** | 0.64 | 0.0% |  |
| NR | 1 | 6.10 (3.12, 9.08) | **<0.001** | - | - |  |
| Health status | | | | | | |
| T2DM | 1 | -9.50 (-17.78 -1.23) | **0.02** | - | - | **0.009** |
| Overweight/Obesity | 3 | 3.41 (-1.97, 8.79) | 0.21 | **0.03** | 73.0% |  |
| Others | 1 | -7.00 (-13.34, -0.65) | **0.03** | - | - |  |
| Baseline BMI (kg/m^2^) | | | | | | |
| 25-29.9 | 3 | -1.95 (-11.38, 7.49) | 0.69 | **0.001** | 85.3% | **0.001** |
| >30 | 1 | 6.10 (3.12, 9.08) | **<0.001** | - | - |  |
| NR | 1 | -9.50 (-17.77, -1.23) | **0.02** | - | - |  |
| Subgroup analyses of Glucomannan on DBP (mmHg) | | | | | | |
| Overall effect | 5 | 3.83 (-0.81, 8.47) | 0.11 | **<0.001** | 88.5% |  |
| Country | | | | | | |
| USA | 1 | 7.80 (3.92, 11.68) | **<0.001** | - | - | **<0.001** |
| Canada | 2 | -1.63 (-4.01, 0.75) | 0.17 | 0.97 | 0.0% |  |
| Others | 2 | 7.62 (5.32, 9.92) | **<0.001** | 0.78 | 0.0% |  |
| Study design | | | | | | |
| Paralell | 3 | 7.67 (5.69, 9.65) | **<0.001** | 0.96 | 0.0% | **<0.001** |
| Cross-over | 2 | -1.63 (-4.02, 0.75) | 0.18 | 0.97 | 0.0% |  |
| Gender | | | | | | |
| Both sexes | 4 | 2.84 (-2.63, 8.33) | 0.30 | **<0.001** | 90.0% | 0.14 |
| Male | 1 | 7.80 (3.91, 11.68) | **<0.001** | - | - |  |
| Age | | | | | | |
| ≤50 | 2 | 7.66 (5.69, 9.64) | **<0.001** | 0.95 | 0.0% | **<0.001** |
| >50 | 3 | -1.63 (-4.01, 0.75) | 0.17 | 0.96 | 0.0% |  |
| Trial duration (weeks) | | | | | | |
| ≤4 | 2 | -1.63 (-4.01, 0.75) | 0.17 | 0.96 | 0.0% | **<0.001** |
| >4 | 3 | 7.66 (5.69, 9.64) | **<0.001** | 0.95 | 0.0% |  |
| Intervention dose (mg/day) | | | | | | |
| ≤3000 | 2 | 7.49 (4.45, 10.53) | **<0.001** | 0.80 | 0.0% | **<0.001** |
| >3000 | 2 | -1.63 (-4.02, 0.75) | 0.18 | 0.97 | 0.0% |  |
| NR | 1 | 7.80 (5.20, 10.40) | **<0.001** | - | - |  |
| Health status | | | | | | |
| T2DM | 1 | -1.70 (-5.82, 2.42) | 0.42 | - | - | **<0.001** |
| Overweight/Obesity | 3 | 7.67 (5.69, 9.65) | **<0.001** | 0.96 | 0.0% |  |
| Others | 1 | -1.60 (-4.52, 1.32) | 0.28 | - | - |  |
| Baseline BMI (kg/m^2^) | | | | | | |
| 25-29.9 | 3 | 4.24 (-2.38, 10.86) | 0.21 | **<0.001** | 88.8% | **0.001** |
| >30 | 1 | 7.80 (5.20, 10.40) | **<0.001** | - | - |  |
| NR | 1 | -1.70 (-5.82, 2.42) | 0.42 | - | - |  |
| Subgroup analyses of Glucomannan on TG (mg/dl) | | | | | | |
| Overall effect | 10 | 4.76 (-2.30, 11.83) | 0.19 | 0.52 | 0.0% |  |
| Country | | | | | | |
| USA | 2 | 12.94 (-7.81, 33.70) | 0.22 | 0.69 | 0.0% | 0.58 |
| Canada | 3 | 13.17 (-13.47, 39.80) | 0.33 | 0.92 | 0.0% |  |
| Others | 5 | 1.47 (-12.70, 15.64) | 0.84 | 0.16 | 0.39 |  |
| Study design | | | | | | |
| Paralell | 5 | 4.80 (-9.29, 18.89) | 0.50 | 0.13 | 43.3% | 0.89 |
| Cross-over | 5 | 6.23 (-10.93, 23.40) | 0.47 | 0.90 | 0.0% |  |
| Gender | | | | | | |
| Both sexes | 7 | 3.28 (-12.07, 18.64) | 0.68 | 0.30 | 17.0% | 0.85 |
| Female | 1 | 4.15 (-4.93, 13.23) | 0.37 | - | - |  |
| Male | 1 | 10.63 (-12.98, 34.24) | 0.38 | - | - |  |
| NR | 1 | 20.80 (-22.74, 64.34) | 0.35 | - | - |  |
| Trial duration (weeks) | | | | | | |
| ≤4 | 6 | 4.61 (-3.42, 12.64) | 0.26 | 0.96 | 0.0% | 0.95 |
| >4 | 4 | 5.43 (-18.14, 29.00) | 0.65 | 0.07 | 57.4% |  |
| Age | | | | | | |
| ≤50 | 4 | 3.19 (-12.67, 19.05) | 0.69 | 0.09 | 53.9% | 0.76 |
| >50 | 5 | 6.24 (-12.93, 23.41) | 0.48 | 0.91 | 0.0% |  |
| NR | 1 | 20.80 (-22.74, 64.34) | 0.35 | - | - |  |
| Intervention dose (mg/day) | | | | | | |
| ≤3000 | 5 | 6.97 (-1.03, 14.96) | 0.09 | 0.69 | 0.0% | 0.08 |
| >3000 | 4 | 5.58 (-11.76, 22.92) | 0.53 | 0.87 | 0.0% |  |
| NR | 1 | -29.20 (-59.44, 1.04) | 0.06 | - | - |  |
| Health status | | | | | | |
| T2DM | 5 | 8.11 (-8.86, 25.09) | 0.35 | 0.85 | 0.0% |  |
| Overweight/Obesity | 4 | 3.19 (-12.67, 19.05) | 0.69 | 0.09 | 53.9% | 0.91 |
| Others | 1 | 8.85 (-38.33, 56.03) | 0.71 | - | - |  |
| Baseline BMI (kg/m^2^) | | | | | | |
| 25-29.9 | 5 | 9.64 (-4.03, 23.31) | 0.17 | 0.70 | 0.0% | 0.62 |
| >30 | 4 | -2.12 (-25.28, 21.05) | 0.86 | 0.08 | 60.3% |  |
| NR | 2 | 15.51 (-18.62, 49.63) | 0.37 | 0.70 | 0.0% |  |
| Subgroup analyses of Glucomannan on TC (mg/dl) | | | | | | |
| Overall effect | 10 | -7.38 (-16.89, 2.13) | 0.13 | **<0.001** | 78.4% |  |
| Country | | | | | | |
| USA | 2 | 8.88 (-23.77, 41.53) | 0.59 | **0.02** | 82.4% | 0.16 |
| Canada | 3 | -20.23 (-35.52, -4.95) | **0.009** | **0.04** | 67.2% |  |
| Others | 5 | -5.05 (-17.34, 7.24) | 0.42 | **0.001** | 79.3% |  |
| Study design | | | | | | |
| Paralell | 5 | 3.32 (-4.85, 11.49) | 0.43 | 0.12 | 45.5% | **0.001** |
| Cross-over | 5 | -19.84 (-30.01, -9.67) | **<0.001** | 0.06 | 55.7% |  |
| Gender | | | | | | |
| Both sexes | 7 | -12.93 (-23.96, -1.90) | **0.02** | **<0.001** | 75.7% | **0.009** |
| Female | 1 | 2.24 (-6.17, 10.65) | 0.60 | - | - |  |
| Male | 1 | -6.96 (-23.40, 9.47) | 0.41 | - | - |  |
| NR | 1 | 26.40 (4.38, 48.41) | **0.02** | - | - |  |
| Trial duration (weeks) | | | | | | |
| ≤4 | 6 | -15.47 (-27.86, -3.08) | **0.01** | **<0.001** | 80.3% | **0.02** |
| >4 | 4 | 5.08 (-7.60, 17.76) | 0.43 | 0.06 | 59.1% |  |
| Intervention dose (mg/day) | | | | | | |
| ≤3000 | 5 | 3.75 (-6.39, 13.89) | 0.47 | 0.11 | 47.1% | 0.002 |
| >3000 | 4 | -21.97 (-33.20, -10.74) | **<0.001** | 0.06 | 60.3% |  |
| NR | 1 | -1.54 (-10.54, 7.46) | 0.74 | - | - |  |
| Health status | | | | | | |
| T2DM | 5 | -9.56 (-25.80, 6.68) | 0.24 | **<0.001** | 81.0% | 0.002 |
| Overweight/Obesity | 4 | 0.45 (-5.10, 5.99) | 0.87 | 0.50 | 0.0% |  |
| Others | 1 | -30.93 (-47.86, -14.00) | 0.24 | - | - |  |
| Baseline BMI (kg/m^2^) | | | | | | |
| 25-29.9 | 5 | -13.31 (-27.90, 1.28) | 0.07 | 0.005 | 73.4% | 0.30 |
| >30 | 3 | -0.99 (-6.49, 4.52) | 0.72 | 0.48 | 0.0% |  |
| NR | 2 | -0.10 (-50.97, 50.77) | 0.99 | **<0.001** | 92.7% |  |
| Subgroup analyses of Glucomannan on LDL-C (mg/dl) | | | | | | |
| Overall effect | 10 | -7.39 (-17.89, 3.10) | 0.17 | **<0.001** | 83.6% |  |
| Country | | | | | | |
| USA | 2 | 5.59 (-22.40, 33.57) | 0.70 | **0.04** | 76.2% | 0.12 |
| Canada | 3 | -20.46 (-33.71, -7.20) | **0.002** | 0.06 | 64.6% |  |
| Others | 5 | -3.87 (-18.58, 10.83) | 0.61 | **<0.001** | 86.4% |  |
| Study design | | | | | | |
| Paralell | 5 | 5.53 (-2.52, 13.59) | 0.18 | 0.15 | 40.5% | **<0.001** |
| Cross-over | 5 | -20.22 (-30.31, -10.12) | **<0.001** | **0.02** | 64.9% |  |
| Gender | | | | | | |
| Both sexes | 7 | -11.86 (-25.58, 1.86) | 0.09 | **<0.001** | 86.4% | 0.07 |
| Female | 1 | 0.96 (-7.84, 9.76) | 0.83 | - | - |  |
| Male | 1 | -7.73 (-24.00, 8.55) | 0.35 | - | - |  |
| NR | 1 | 20.90 (-1.12, 42.92) | 0.06 | - | - |  |
| Trial duration (weeks) | | | | | | |
| ≤4 | 6 | -16.30 (-27.94, -4.66) | **0.006** | **<0.001** | 81.2% | **0.003** |
| >4 | 4 | 7.73 (-3.19, 18.65) | 0.17 | 0.16 | 42.7% |  |
| Intervention dose (mg/day) | | | | | | |
| ≤3000 | 5 | 0.68 (-8.24, 9.61) | 0.88 | 0.15 | 41.5% | **<0.001** |
| >3000 | 4 | -23.17 (-33.88, -12.46) | **<0.001** | 0.05 | 60.8% |  |
| NR | 1 | 10.43 (1.10, 19.76) | **0.03** | - | - |  |
| Health status | | | | | | |
| T2DM | 5 | -11.44 (-25.67, 2.79) | 0.12 | **0.001** | 79.1% | **<0.001** |
| Overweight/Obesity | 4 | 3.79 (-4.01, 11.58) | 0.34 | 0.20 | 35.3% |  |
| Others | 1 | -30.93 (-44.48, -17.38) | **<0.001** | - | - |  |
| Baseline BMI (kg/m^2^) | | | | | | |
| 25-29.9 | 5 | -15.08 (-29.05, -1.11) | **0.03** | **0.002** | 76.4% | 0.16 |
| >30 | 3 | 1.72 (-8.19, 11.63) | 0.73 | 0.05 | 64.7% |  |
| NR | 2 | -1.58 (-44.00, 40.84) | 0.94 | **0.001** | 90.3% |  |
| Subgroup analyses of Glucomannan on HDL-C (mg/dl) | | | | | | |
| Overall effect | 9 | 0.60 (-0.44, 1.64) | 0.26 | 0.94 | 0.0% |  |
| Country | | | | | | |
| USA | 1 | -1.94 (-7.59, 3.71) | 0.50 | - | - | 0.55 |
| Canada | 3 | -0.15 (-3.00, 2.70) | 0.92 | 0.86 | 0.0% |  |
| Others | 5 | 0.83 (-0.31, 1.97) | 0.16 | 0.84 | 0.0% |  |
| Study design | | | | | | |
| Paralell | 4 | 0.95 (-0.29, 2.19) | 0.13 | 0.69 | 0.0% | 0.32 |
| Cross-over | 5 | -0.22 (-2.13, 1.69) | 0.82 | 0.98 | 0.0% |  |
| Gender | | | | | | |
| Both sexes | 7 | 0.27 (-1.18, 1.73) | 0.71 | 0.96 | 0.0% | 0.48 |
| Female | 1 | 1.16 (-0.38, 2.70) | 0.50 | - | - |  |
| Male | 1 | -1.94 (-7.59, 3.71) | 0.14 | - | - |  |
| Age | | | | | | |
| ≤50 | 4 | 0.95 (-0.29, 2.18) | 0.13 | 0.69 | 0.0% | 0.32 |
| >50 | 5 | -0.22 (-2.13, 1.64) | 0.82 | 0.98 | 0.0% |  |
| Trial duration (weeks) | | | | | | |
| ≤4 | 6 | 0.62 (-0.58, 1.82) | 0.31 | 0.89 | 0.0% | 0.96 |
| >4 | 3 | 0.56 (-1.53, 2.64) | 0.60 | 0.53 | 0.0% |  |
| Intervention dose (mg/day) | | | | | | |
| ≤3000 | 4 | 0.72 (-0.69, 2.13) | 0.32 | 0.58 | 0.0% | 0.72 |
| >3000 | 4 | -0.08 (-2.13, 1.97) | 0.94 | 0.96 | 0.0% |  |
| NR | 1 | 1.16 (-1.17, 3.49) | 0.33 | - | - |  |
| Health status | | | | | | |
| T2DM | 4 | -0.24 (-2.23, 1.76) | 0.82 | 0.93 | 0.0% |  |
| Overweight/Obesity | 4 | 0.95 (-0.29, 2.19) | 0.13 | 0.68 | 0.0% | 0.60 |
| Others | 1 | 0.00 (-6.77, 6.77) | 0.99< | - | - |  |
| Baseline BMI (kg/m^2^) | | | | | | |
| 25-29.9 | 5 | -0.58 (-2.72, 1.56) | 0.60 | 0.97 | 0.0% | 0.32 |
| >30 | 3 | 1.08 (-0.14, 2.29) | 0.08 | 0.93 | 0.0% |  |
| NR | 1 | -1.55 (-7.37, 4.27) | 0.60 | - | - |  |

**Abbreviations:** CI, confidence interval; WMD, weighted mean differences; NR, not reported; BMI, body mass index; BFP, body fat percentage; FBG, fasting blood glucose; TG, triglyceride; TC, total cholesterol; LDL, low-density lipoprotein; HDL, high-density lipoprotein; SBP, systolic blood pressure; DBP, diastolic blood pressure; T2DM, type 2 diabetes mellitus.

^*^Bold indicates statistical significance

**Supplementary Table 3.** Sensitivity Analysis of the Pooled Effect Sizes Using an Alternative Within-Participant Correlation Coefficient (R = 0.5) for Cross-Over Trials and (R = 0.8) for parallel trials.

|  | NO | WMD (95%CI) | P-value | | heterogeneity | | | |
| --- | --- | --- | --- | --- | --- | --- | --- | --- |
|  |  |  |  |  | P heterogeneity | I^2^ | | P between sub-groups |
| Subgroup analyses of Glucomannan on weight (Kg) | | |  | |  |  | |  |
|  | 9 | 0.58 (-0.73, 1.88) | 0.39 | | 0.21 | 26.7% | |  |
| Subgroup analyses of Glucomannan on BMI (Kg/m^2^) | | | | | | | | |
| Overall effect | 4 | -0.30 (-2.39, 1.79) | 0.78 | | **0.004** | 77.5% | |  |
| Subgroup analyses of Glucomannan on BFP (%) | | | | | | | | |
| Overall effect | 3 | -1.03 (-2.30, 0.23) | 0.11 | | 0.22 | 34.0% | |  |
| Subgroup analyses of Glucomannan on FBG (mg/dl) | | | | | | | | |
| Overall effect | 7 | -3.53 (-8.35, 1.29) | | 0.15 | 0.05 | | 52.0% |  |
| Subgroup analyses of Glucomannan on Insulin (uU/mL) | | | | | | | | |
| Overall effect | 3 | -0.55 (-2.79, 1.69) | 0.63 | | 0.98 | 0.0% | |  |
| Subgroup analyses of Glucomannan on SBP (mmHg) | | | | | | | | |
| Overall effect | 5 | -0.35 (-6.32, 5.63) | 0.91 | | **0.001** | 78.1% | |  |
| Subgroup analyses of Glucomannan on DBP (mmHg) | | | | | | | | |
| Overall effect | 5 | 4.20 (0.07, 8.32) | 0.046 | | **0.001** | 79.8% | |  |
| Subgroup analyses of Glucomannan on TG (mg/dl) | | | | | | | | |
| Overall effect | 10 | 4.62 (-2.81, 12.06) | 0.22 | | 0.58 | 0.0% | |  |
| Subgroup analyses of Glucomannan on TC (mg/dl) | | | | | | | | |
| Overall effect | 10 | -5.29 (-14.30, 3.72) | 0.25 | | **0.003** | 63.9% | |  |
| Subgroup analyses of Glucomannan on LDL-C (mg/dl) | | | | | | | | |
| Overall effect | 10 | -5.81 (-15.79, 4.16) | 0.25 | | **<0.001** | 72.7% | |  |
| Subgroup analyses of Glucomannan on HDL-C (mg/dl) | | | | | | | | |
| Overall effect | 9 | 0.78 (-0.37, 1.92) | 0.18 | | 0.98 | 0.0% | |  |

**Abbreviations:** CI, confidence interval; WMD, weighted mean differences; NR, not reported; BMI, body mass index; BFP, body fat percentage; FBG, fasting blood glucose; TG, triglyceride; TC, total cholesterol; LDL, low-density lipoprotein; HDL, high-density lipoprotein; SBP, systolic blood pressure; DBP, diastolic blood pressure; T2DM, type 2 diabetes mellitus.

^*^Bold indicates statistically significant

**Supplementary Table** **4**. GRADE profile of glucomannan consumption for cardiometabolic parameters.

| **Outcomes** | **Risk of bias** | **Inconsistency** | **Indirectness** | **Imprecision** | **Publication Bias** | **Quality**  **of evidence** |
| --- | --- | --- | --- | --- | --- | --- |
| **Weight** | No serious limitations | No serious limitations | No serious limitations | Serious limitations ^b^ | No serious limitations | ⊕⊕⊕◯ Moderate |
| **BMI** | No serious limitations | Very serious limitations ^a^ | No serious limitations | Serious limitations ^b^ | No serious limitations | ⊕◯◯◯ Very low |
| **BFP** | No serious limitations | No serious limitations | No serious limitations | Serious limitations ^b^ | No serious limitations | ⊕⊕⊕◯ Moderate |
| **FBG** | No serious limitations | Very serious limitations ^a^ | No serious limitations | No serious limitations | No serious limitations | ⊕⊕◯◯  Low |
| **Insulin** | No serious limitations | No serious limitations | No serious limitations | Serious limitations ^b^ | No serious limitations | ⊕⊕⊕◯ Moderate |
| **SBP** | No serious limitations | Very serious limitations ^a^ | No serious limitations | Serious limitations ^b^ | Serious limitations ^c^ | ⊕◯◯◯ Very low |
| **DBP** | No serious limitations | Very serious limitations ^a^ | No serious limitations | Serious limitations ^b^ | No serious limitations | ⊕◯◯◯ Very low |
| **TG** | No serious limitations | No serious limitations | No serious limitations | Serious limitations ^b^ | No serious limitations | ⊕⊕⊕◯ Moderate |
| **TC** | No serious limitations | Very serious limitations ^a^ | No serious limitations | Serious limitations ^b^ | No serious limitations | ⊕◯◯◯ Very low |
| **LDL-C** | No serious limitations | Very serious limitations ^a^ | No serious limitations | Serious limitations ^b^ | No serious limitations | ⊕◯◯◯ Very low |
| **HDL-C** | No serious limitations | No serious limitations | No serious limitations | Serious limitations ^b^ | serious limitations ^c^ | ⊕⊕◯◯  Low |

Abbreviations: BMI, body mass index; BFP, body fat percentage; FBG, fasting blood glucose; TG, triglycerides; TC, total cholesterol; LDL, low-density lipoprotein; HDL, high-density lipoprotein; SBP, systolic blood pressure; DBP, diastolic blood pressure.

a) There was a significant high heterogeneity (I^2^ > 75%).

b) There is no evidence of significant impact of glucomannan intake.

c) There was a significant publication bias based on Egger’s test.

**Supplementary Figure 1.** Funnel plots for the effect of Glucomannan on (A) body weight (kg); (B) BMI (kg/m^2^); (C) BFP (%); (D) SBP (mmHg); (E) DBP (mmHg), (F) FBG (mg/dL), (G) insulin (uU/mL), (H) TG (mg/dL), (I) TC (mg/dL), (J) LDL-C (mg/dL), and (K) HDL-C (mg/dL).

Figure. 1A.

Figure 1B.

Figure 1C.

Figure 1D.

Figure 1E.

Figure 1F.

Figure 1G.

Figure 1H.

Figure 1I.

Figure 1J.

Figure 1K.
